# An *Arabidopsis* Example of Association Mapping in Structured Samples

**DOI:** 10.1371/journal.pgen.0030004

**Published:** 2007-01-19

**Authors:** Keyan Zhao, María José Aranzana, Sung Kim, Clare Lister, Chikako Shindo, Chunlao Tang, Christopher Toomajian, Honggang Zheng, Caroline Dean, Paul Marjoram, Magnus Nordborg

**Affiliations:** 1 Molecular and Computational Biology, University of Southern California, Los Angeles, California, United States of America; 2 Department of Cell and Developmental Biology, John Innes Centre, Norwich, United Kingdom; 3 Department of Preventive Medicine, Keck School of Medicine, University of Southern California, Los Angeles, California, United States of America; North Carolina State University, United States of America

## Abstract

A potentially serious disadvantage of association mapping is the fact that marker-trait associations may arise from confounding population structure as well as from linkage to causative polymorphisms. Using genome-wide marker data, we have previously demonstrated that the problem can be severe in a global sample of 95 Arabidopsis thaliana accessions, and that established methods for controlling for population structure are generally insufficient. Here, we use the same sample together with a number of flowering-related phenotypes and data-perturbation simulations to evaluate a wider range of methods for controlling for population structure. We find that, in terms of reducing the false-positive rate while maintaining statistical power, a recently introduced mixed-model approach that takes genome-wide differences in relatedness into account via estimated pairwise kinship coefficients generally performs best. By combining the association results with results from linkage mapping in F2 crosses, we identify one previously known true positive and several promising new associations, but also demonstrate the existence of both false positives and false negatives. Our results illustrate the potential of genome-wide association scans as a tool for dissecting the genetics of natural variation, while at the same time highlighting the pitfalls. The importance of study design is clear; our study is severely under-powered both in terms of sample size and marker density. Our results also provide a striking demonstration of confounding by population structure. While statistical methods can be used to ameliorate this problem, they cannot always be effective and are certainly not a substitute for independent evidence, such as that obtained via crosses or transgenic experiments. Ultimately, association mapping is a powerful tool for identifying a list of candidates that is short enough to permit further genetic study.

## Author Summary

There is currently tremendous interest in using association mapping to find the genes responsible for natural variation, particularly for human disease. In association mapping, researchers seek to identify regions of the genome where individuals who are phenotypically similar (e.g., they all have the same disease) are also unusually closely related. A potentially serious problem is that spurious correlations may arise if the population is structured so that members of a subgroup tend to be much more closely related. We have previously demonstrated that this problem can be severe in *Arabidopsis thaliana,* and that established statistical methods for controlling for population structure are insufficient. Here, we evaluate a broader range of methods. We find that a recently introduced mixed-model approach generally performs best. By combining the association results with results from linkage mapping in F2 crosses, we identify one previously known true positive and several promising new associations, but also demonstrate the existence of both false positives and false negatives. Our results illustrate the potential of genome-wide association scans as a tool for dissecting the genetics of natural variation, while at the same time highlighting the pitfalls.

## Introduction

As sequencing and genotyping costs continue to decrease, association mapping (also known as linkage disequilibrium mapping) is emerging as a powerful, general tool for identifying alleles and loci responsible for natural variation. Although its application to human disease has received most attention [1], association mapping has tremendous potential in organisms ranging from Arabidopsis thaliana [2] to cattle [3]. Indeed, given how quickly sequencing and genotyping technology is developing, it will soon be feasible to carry out genome-wide association scans in almost any organism, including many that are not amenable to traditional mapping methods (e.g., because they cannot be crossed experimentally).

A potentially serious obstacle to association mapping is confounding by population structure. It is well known that population structure may cause spurious correlations, leading to an elevated false-positive rate [4]. Whether this is likely to be a significant problem in human association studies is currently a subject of considerable debate [5–11]. Be that as it may, human association studies are typically designed as case-control studies, and the effects of confounding can thus be minimized by carefully matching cases and controls [7]. When association mapping is instead used to identify genes responsible for quantitative variation in a single population sample, there is every reason to believe that confounding will be a significant problem, especially if the trait varies geographically, as does height, skin color, or flowering time [7,12–14].

In an earlier paper, we demonstrated that confounding can be severe in a structured sample of 95 A. thaliana accessions for which genome-wide polymorphism data are available [13]. We also showed that the two best-known statistical approaches for dealing with this type of confounding, “genomic control” [15] and “structured association” [16], were not effective, the former because it lacks power in the presence of strong confounding, the latter because it did not adequately capture the complex pattern of relatedness in the sample. Although we were able to identify several previously known loci, the false-positive rate remained sufficiently elevated to make searching for new loci infeasible. In this paper, we evaluate a wider range of methods, including the mixed-model approach of Yu et al. [14] and the principal components approach of Price et al. [17]. We first investigate how these methods perform in terms of reducing the false-positive rate in genome-wide scans for a range of flowering-related phenotypes, then examine their power using data-perturbation simulations.

We find that versions of the approach of Yu et al. generally perform best, but a comparison with the results of linkage mapping indicates that some confounding still remains, and, furthermore, that correcting for population structure also introduces false negatives. Nonetheless, the false-positive rate is reduced to levels that make it possibly to use our sample for association mapping, and although our scans have low power due to insufficient marker density and small sample size, we identify a number of plausible associations.

## Results

### Confounding by Population Structure

Mean flowering time for each accession was measured under a variety of experimental conditions, at the University of Southern California (USC) and at the John Innes Centre (JIC). In addition, we measured expression levels of two key flowering time genes: the floral repressor *FLC* [19,20] and its promoter gene *FRI* [21]. The phenotypes used are summarized in Table 1 (and are available as Dataset S1). The genotypes were in the form of over 900 short sequenced fragments, distributed throughout the genome [22]. Depending on the analysis, each sequenced fragment was either broken up into constituent single nucleotide polymorphisms (SNPs), or treated as a multi-allelic locus (with alleles corresponding to unique haplotypes after removing singleton SNPs). The genotypes are available as Dataset S2.

**Table 1:**
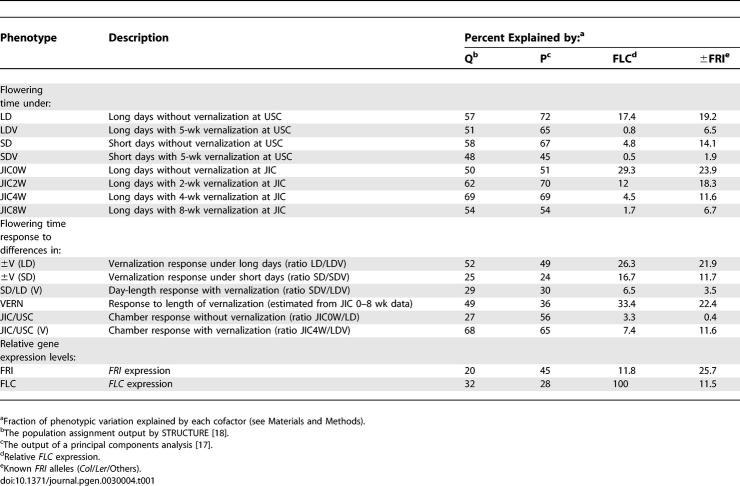
Summary of Phenotypes Used

**Figure 1:**
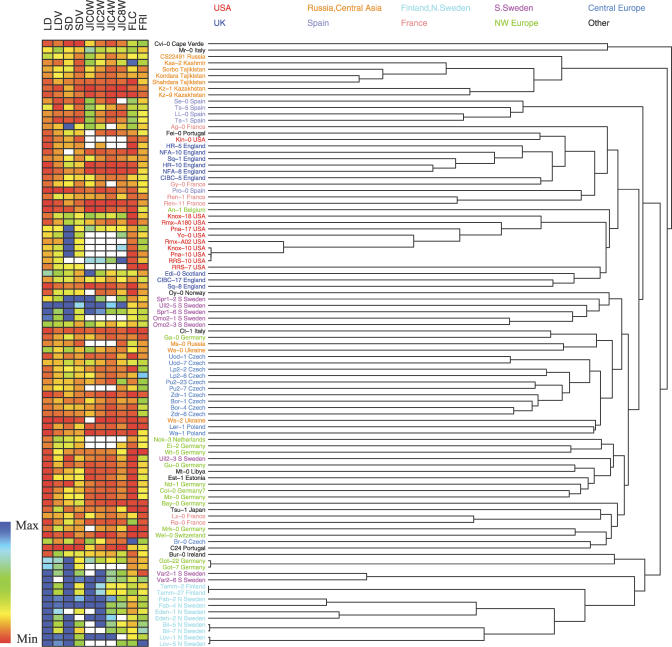
Summary of the Data Illustrating Strong Positive Correlations between Phenotypes and between Phenotypes and Genome-Wide Relatedness The panel on the left gives the basic phenotypes used (see Table 1), with colors indicating relative values within each phenotype (white denotes missing data). The tree on the right shows a hierarchical clustering (UPGMA) of accessions based on their relative kinship (as measured by pairwise haplotype sharing). Colors for the accessions labels indicate geographic origins.

The phenotypes were generally strongly correlated across accessions (Figure 1). As expected given our previous results, they were also correlated with the genome-wide pattern of relatedness (summarized using a tree in Figure 1), indicating that the null hypothesis of no association between genotype and phenotype is false in a genomic sense, and that the false-positive rate must therefore be inflated by spurious correlations [13].

**Figure 2:**
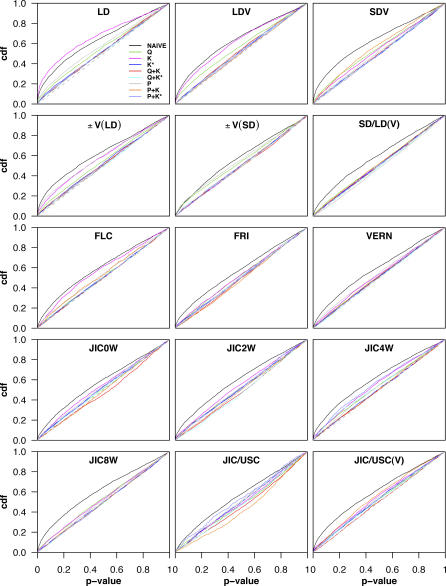
The Cumulative Distribution of *p*-Values in Genome-Wide Scans for All Phenotypes The different curves correspond to different approaches for correcting for population structure (described in Table 2). Without correction for population structure, all distributions are strongly skewed towards significance. The results shown here are for associations between phenotype and individual SNPs: results for haplotypes were very similar (see Figure S2). cdf, cumulative distribution function

And inflated it is. Figure 2 shows the cumulative distribution of *p*-values from association scans across all loci for all our phenotypes. The black curves correspond to naive tests of association without correction for population structure. Irrespective of phenotype, the distribution was strongly skewed towards significance, in agreement with our previous observations [13].

**Table 2:**
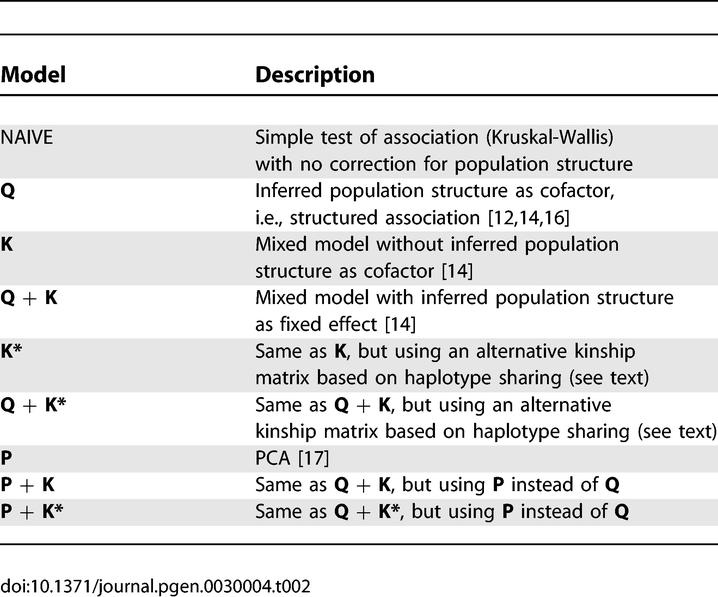
Summary of Models Used

### Statistical Approaches for Reducing Confounding

Pritchard et al. [16] introduced so-called “structured association” for reducing confounding due to population structure. The approach is based on first assigning individuals to subpopulations using a model-based Bayesian clustering algorithm, STRUCTURE, and then carrying out all analyses conditional on the inferred assignments. To the extent that it is possible to capture the relevant structure this way, the approach is eminently sensible, but as we have reported earlier, it does not appear to be sufficient for the sample used here [13]. The same was true in general for the present study, although performance varied greatly between phenotypes, and was in fact reasonable in a couple of cases.

Yu et al. [14] recently introduced a mixed-model approach to control for population structure. The key to their approach is using a random effect to estimate the fraction of the phenotypic variation that can be explained by genome-wide correlations. The individual random deviations from the population mean are constrained by assuming that the (phenotypic) covariance between individuals is proportional to their relative relatedness (or kinship), which is estimated using genome-wide marker data. In addition to this random effect, Yu et al. used the population assignments produced by the STRUCTURE algorithm (the **Q** matrix) as a fixed effect in the model, as in structured association [12,14,16].

We applied the approach of Yu et al. [14] to analyze our data. We found that their full model (combining kinship and structured association) successfully reduced the false-positive rate almost to expected levels, indicating that confounding by population structure has largely been eliminated (Figure 2).

The mixed model of Yu et al. includes two terms that are intended to accomplish the same thing. Population structure is modeled both as a fixed effect (using the **Q** matrix) and as a random effect (using the **K** matrix of pairwise kinship coefficients) [14]. While it is intuitively obvious that the latter captures features of the data that could not possibly be captured by the former (e.g., different levels of relatedness; see Discussion), it is by no means clear that the **Q** matrix should be required in addition to the kinship effect. Nonetheless, we found that it is (i.e., the **K** matrix was not sufficient; see Figure 2), as did Yu et al. [14].

We suspected that this might be an artifact of how kinship was estimated. The formula used by Yu et al. [14] is based on classical work on estimating kinship, defined as the fraction of the genome shared identical by descent, using marker data [23–25]. A distinction is made in this work between identity by descent and identity in state, where the latter is defined, loosely speaking, as the amount of allele sharing expected between “unrelated” individuals. This would seem to make little sense in the context of association mapping: there are no unrelated individuals [26], and identity in state implies identity by descent when using markers with a very low mutation rate (such as SNPs). It seemed to us that kinship should simply be estimated as the fraction of shared alleles.

We tried this, and found that, indeed, with this new measure of kinship, the false-positive rate could often (but not always) be reduced almost as well by using kinship alone. It turns out that the best results were obtained not by considering the fraction of shared SNP alleles, but by considering the fraction of shared fragment haplotypes (recall that our data are in the form of short sequence fragments), presumably because these capture the underlying structure better. As illustrated in Figure 2, correcting for population structure using our simpler kinship estimate often worked as well as using the full model of Yu et al. [14] in terms of reducing the false-positive rate. Estimating population structure using STRUCTURE thus appears to be unnecessary in many cases. Yu et al. have independently reached similar conclusions (E. S. Buckler, G. Pressoir, J. Yu, Z. Zhang, unpublished data). However, for some phenotypes the false-positive rate was still clearly inflated, and we therefore also analyzed the data using both the **Q** matrix and our new kinship estimate as cofactors (see Table 2). The results from this model were very similar to those obtained using the full model of Yu et al.

One reason for investigating methods that do not require the use of the **Q** matrix is that the STRUCTURE algorithm is computationally intensive, and may be impractical on large datasets. With this in mind, Price et al. [17] suggested that a principal components analysis (PCA) be used to summarize genome-wide patterns of relatedness instead of the **Q** matrix. We tried this approach as well, and found that although it performed better than using **Q** alone, it did not perform as well as our modified kinship matrix, nor the approaches that combine kinship estimates with **Q**. Finally, we tried a mixed-model approach that combined the PCA assignments (the **P** matrix) with kinship estimates. This approach performed similarly to mixed-models using **Q**, suggesting that it may be possible to replace the computationally intensive STRUCTURE algorithm with a simple PCA in the mixed-model approach.

### The Effect on Power

The relative power of the various approaches was compared using data-perturbation simulations. Large numbers of perturbed datasets were generated by assigning a phenotypic effect (expressed as a deviation from the population mean) to randomly chosen SNPs (one per simulated dataset). These datasets were then analyzed using the various approaches, and, for each approach, we recorded the fraction of perturbed datasets for which the *p*-value for the simulated quantitative trait nucleotide (QTN) was in the 5th percentile (estimated from the genome-wide SNPs).

**Figure 3:**
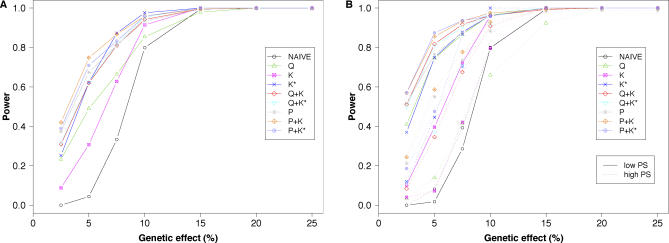
Power of the Different Methods to Detect a QTN The power of the different methods to detect a QTN using a 5% significance level (based on the observed distribution of *p*-values), as a function of the fraction of the phenotypic variation attributable to the QTN. (A) Power averaged across all simulated QTN. Methods that reduce confounding more effectively have better power. (B) Power calculated separately for QTN that showed strong correlation with population structure (“high PS,” arbitrarily defined as simulations where more than 50% of the phenotypic variation could be explained by **Q** (note that “**P**” performs better than “**Q**” largely because of this definition) and those that did not (“low PS”). All methods are relatively powerless to detect QTN that are strongly correlated with population structure. Results for haplotypes were similar (Figure S3).

Figure 3A shows the resulting power estimates as a function of the fraction of the phenotypic variation explained by the QTN. In general, approaches that reduced confounding more effectively had better power than approaches that did not, presumably because the confounding noise tended to overwhelm the signal of the true association in the latter cases.

While these results might seem to indicate that it is possible to reduce the false-positive rate without paying a price in terms of an increased false-negative rate (i.e., decreased power), this is by no means the case. All the methods used here attempt to reduce confounding by identifying and eliminating genotype–phenotype associations that are in effect genome-wide because they reflect underlying population structure, leaving only those associations that cannot be accounted for by this structure. However, not all the associations thus eliminated will be false; indeed, in a genome-wide scan some of them will almost certainly be true [13]. To give an extreme example, if the causal polymorphisms are perfectly correlated with the underlying population structure (e.g., because of selection), it will not be possible to distinguish between true and false positives statistically, and any attempt to remove the latter will remove the former. This effect could also be seen in our power study when we separated the simulated QTN into those that happened to be strongly associated with population structure and those that did not. As shown in Figure 3B, power to detect the former was considerably poorer than power to detect the latter.

### Residual Confounding

Since several of the methods discussed above produced *p*-value distributions that looked reasonable, we proceeded to analyze the results of our genome-wide scan using the methods that produced reasonably flat *p*-value distributions. For these analyses, we decided to focus on treating our sequence data as short haplotypes, partly because we expect power to be higher when the information inherent in linkage disequilibrium between SNPs is utilized [27], partly to reduce the problem of multiple comparisons (which also reduces power). Table 3 lists the number of loci that are significant using a nominal 0.1% level for each phenotype and each method (for detailed output of the scans, see Dataset S3 and Figures S4–S8). The number of significant loci under the naive Kruskal-Wallis approach is included for comparison. It is clear that there were many more significant associations than could be explained by chance alone. For several reasons, we believe that many, if not most, of these are spurious correlations due to residual confounding rather than true associations.

**Table 3:**
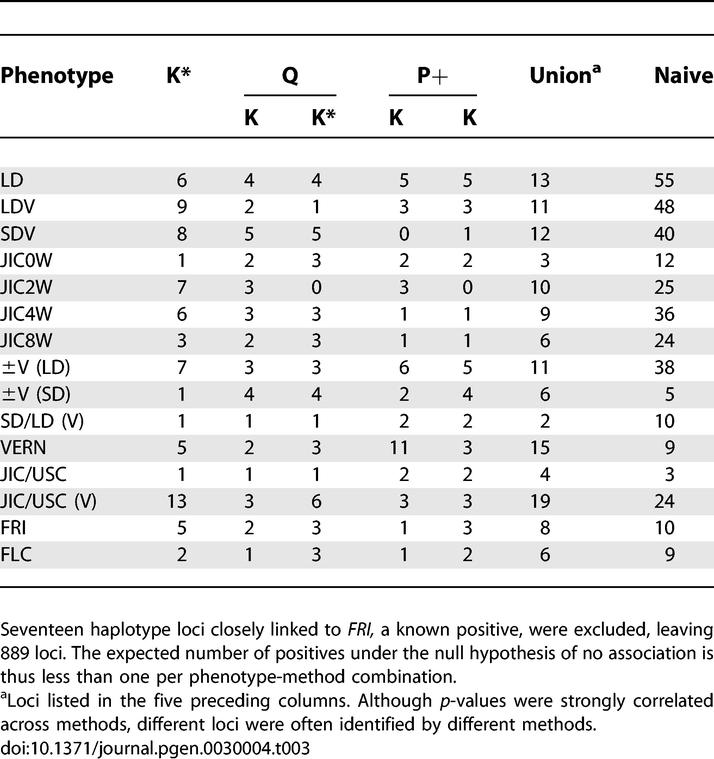
Number of Significant Loci at a Nominal 0.1% Level

**Figure 4:**
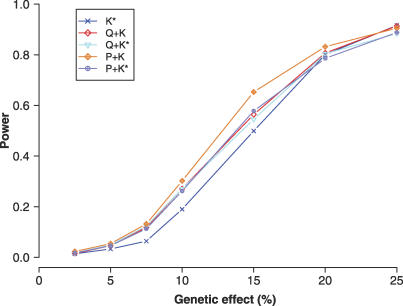
Power to Detect Associations Using a Nominal 0.1% Significance Level The QTN is assumed to lie within a sequenced haplotype, but power is nonetheless poor even for major QTL.

The first reason for skepticism is general pessimism about the power of the study. In other words, we would not have expected to find such a high number of true positives. This argument relies partly on a priori notions about the genetic architecture of the trait (in particular about genetic heterogeneity), and partly on demonstrable limitations of the study. Our study is clearly underpowered in two different ways [13]. First, the sample is too small to detect anything but very major quantitative trait loci (QTL) even if we assume that the causal polymorphisms themselves have been included in the sequencing. Figure 4 shows the expected power to detect such loci (at a nominal significance level of 0.1%) as estimated from our data perturbation simulations. To put these results in perspective, note that the major loss-of-function alleles at the vernalization-response locus *FRI* explained between 14% and 24% of the variation for flowering time in the absence of a vernalization treatment (Table 1). Expected power to detect a locus like *FRI,* by most standards a major QTL, would thus be 40%–80% in our sample. Second, the marker density is insufficient given the extent of linkage disequilibrium in the sample. It is difficult to quantify the resulting loss in power because linkage disequilibrium is so variable, but an optimistic estimate is that 20% of the genome is effectively being screened [13]. Putting these results together, it is clear that we should not expect to detect more than a minor fraction of major genes. If we believe that all the associations in Table 3 are real, we must thus also believe that there are at least an order of magnitude more major loci that have not been detected. As we shall see below, such a belief is contradicted by data.

The second reason for skepticism is that the associations observed strongly suggest residual confounding. We have previously noted that many of the strongest associations seem to be due to allele sharing among very late-flowering accessions from Finland and northern Sweden [13,27]. These associations have largely been removed by the methods used here, but probably not completely. Many of the remaining significant associations in this study involved small clusters of these late-flowering accessions with a few others thrown in. It is likely that the correlation with population structure for these clusters was not sufficiently strong for the various methods to eliminate them.

**Figure 5:**
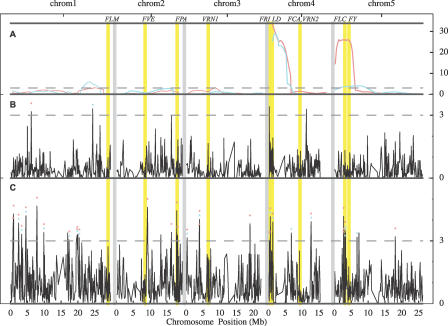
Comparison of Association and Linkage Mapping Results for the Phenotype JIC4W The position of candidate genes with higher marker density are highlighted in yellow. (A) The QTL LOD scores for two crosses: *Col-0* × *Lov-1* (pink curve) and *Col-0* × *Edi-0* (cyan curve). The dashed grey line marks a genome-wide permutation significance level of 5%. (B) Negative log *p*-values from a genome-wide scan using the **Q** + **K** model. (C) Negative log *p*-values from a genome-wide scan using a naive Kruskal-Wallis approach. (B and C) The dashed grey line marks a nominal significance level of 0.1%, and the colored stars denote associations that should correspond to QTL in the cross displayed using the same color (because the appropriate alleles are segregating; see text).

The third and strongest reason for skepticism is direct comparison with linkage mapping results [28]. Figure 5 shows a comparison between the genome-wide association scans for flowering time after four weeks vernalization at the John Innes Centre, and the linkage mapping experiments undertaken under similar conditions (Figures S9 and S10 show the results of different crosses, undertaken at zero and eight weeks vernalization, respectively). The existence of a (true) association at a particular locus implies that a QTL overlapping this locus should exist in crosses involving accessions that carry different alleles for the locus in question (the reverse is not true; the existence of a QTL in cross does not imply the existence of a population association because the QTL could be due to a rare allele with negligible effect on the population variation). Associations that ought to correspond to a QTL in a particular cross have been labeled accordingly in Figure 5. The overall lack of correspondence between the association and linkage mapping results is obvious. One of the surprising results of the linkage mapping experiments was that, across six crosses, almost all of the variation was due to QTL in three genomic regions: the top of Chromosome 4 (certainly involving *FRI,* and possibly also other loci), the top of Chromosome 5 (with *FLC* as a strong candidate for at least part of the effect), and the bottom of Chromosome 1 (certainly involving multiple QTL). There was no evidence of any QTL on Chromosomes 2 or 3. Nominally significant associations, in contrast, although far from uniformly distributed, were found across the genome, indicating that most are likely to be spurious even after confounding has been reduced statistically. Note that this argument relies on the linkage mapping experiments having sufficient power; however, it seems unlikely that a QTL would be detectable as an association in a heterogeneous sample of 96 accessions and not be detectable in an F2 cross using twice the sample size.

### Plausible Associations

Given that confounding remains, it was clear that the best we could hope to accomplish was compiling a list of plausible associations worthy of further study. How this should be done is a highly subjective matter. We decided to use consistency across association methods as our main criterion. Specifically, we only consider loci significant at the 0.1% level across all five methods in Table 3. This procedure resulted in a short list of seven loci, shown in Table 4.

**Table 4:**
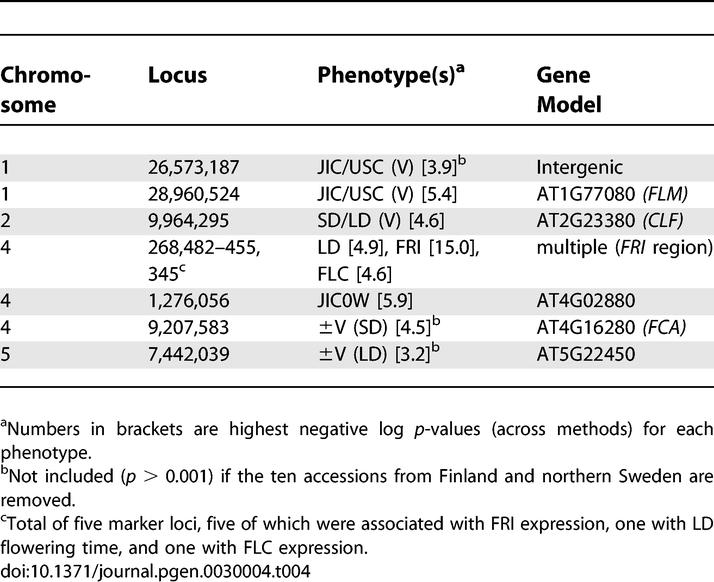
The Most Promising Associations

Several features of Table 4 support the notion that these associations are good candidates for being true associations. First, *FRI,* the one locus known a priori to be involved in flowering time variation, generally shows the pattern of association we would expect it to show. Particularly noteworthy was the incredibly strong association with *FRI* expression levels, almost completely due to the *cis*-regulatory mutation present in *Ler* and 12 other accessions. Allelic variation at *FRI* was also associated with variation in *FLC* expression, in agreement with what is known about the function of these genes [29]. Second, leaving *FRI* aside, two out six of the significant associations occurred in our candidate genes. Since 18 out of 889 marker loci were located in candidate genes, this is sixteen times more than would be expected by chance. Third, one of the four associations not in a gene identified *a priori* as a candidate, was in a gene identified *a posteriori* as a good candidate for being involved in variation for the phenotype with which it was associated (*CLF* and response to differences in day length [30,31]). Finally, note that none of the associations in Table 4 are contradicted by the crosses. The phenotypes are different except for AT4G02880, which is significant for JIC0W (Figure S9); this association coincides with the QTL overlapping *FRI*.

### Published Associations

Several recent papers have reported associations between flowering time and genotypes at candidate loci. As part of a study involving field measurement of flowering times, Olsen et al. [32] and Caicedo et al. [33] found associations with *CRY2* and *FLC,* respectively. They claimed to have controlled for population structure by using 79 AFLP markers to identify an “unstratified” population in which to test for associations. This claim was directly contradicted by the observation that the significant associations reported in these papers showed clear geographic patterns, however, and a simple analysis of the distribution of *p*-values across the AFLP markers reveals a skew similar to the ones we have discussed in this paper (Figure S12).

A separate study by Balasubramanian et al. [34] reported associations with *PHYC*. Although the *p*-value distribution was strongly skewed in this study as well (see their Figure 4), the claim of association was rather more convincing because it was supported by several independent sources of evidence (including linkage mapping results).

We decided to investigate these published polymorphisms in our sample, where the genome-wide data would make it possible to evaluate the significance of any associations. Using the various mixed-model approaches, *PHYC* was marginally significant for ±V (LD) and VERN (*p*-values ranged from 0.01 to 0.07 depending on the method used, see Table S1), while *FLC* showed a tendency towards significance (*p*-values ranged from 0.03 to 0.13) for SD, SD/LD (V), and FLC. *CRY2* showed no sign of being associated with any phenotype. Clearly, none of these polymorphisms would have been picked up in a genome-wide scan.

Naive analyses using standard linear methods without correction for population structure (as in the original papers) often identified what appeared to be highly significant associations. Furthermore, adding *FRI* genotype and latitude as terms to the model (as was done in some of the original papers) often had a dramatic effect on the results. For example, *CRY2* was associated (at a nominal 5% significance level) with LDV, SDV, and JIC8W, but became associated with 11 out of 16 phenotypes if *FRI* genotype was added to the model. The significance of a particular term was often strongly dependent on which other terms were included in the model (Tables S2–S4). This is probably to be expected; in the presence of confounding population structure, including these kinds of terms is essentially a form of partial genomic control, no different from including the **Q** matrix [14], the **P** matrix [17], or selected control SNPs [11].

The nominal *p*-values are of course affected by confounding population structure as discussed earlier in this paper, and we therefore attempted to assess their significance by comparing them to the genome-wide distribution of *p*-values, obtained by testing either all SNPs or short haplotypes in our data using the same model. While this might appear to be a robust procedure guaranteed to yield valid *p*-values, it is in fact anti-conservative because the candidate polymorphisms were ascertained to be informative about local haplotype structure. For example, the SNPs used to “tag” the three *CRY2* “haplogroups” [32] are not random SNPs that can be compared to randomly chosen genome-wide SNPs; they are SNPs that were selected precisely because they identify highly diverged haplotypes. It is reasonable to assume that such SNPs are also more strongly associated with population structure than are random SNPs. By comparing a tag SNP association to the genome-wide distribution based on random SNPs, we are thus likely to exaggerate its significance. Comparing to haplotype markers should help, but there is no guarantee. Even so, we found that most of the nominally significant associations disappear when the genome-wide distribution was used as a control.

Considering the number of phenotypes tested, *CRY2* showed no significant associations when compared to the genome-wide distribution of haplotype tests. There were more significant associations than could be expected when genome-wide SNPs were used as a control, but there are several reasons to be skeptical of these: first, the *CRY2* polymorphism is in fact a haplotype polymorphism with three alleles, and should thus be compared to the haplotype distribution; second, most of the associations disappear if a *CRY2* by *FRI* interaction term is added to the model; and third, at least one of the associations is directly contradicted by our linkage mapping results (different *CRY2* haplotypes segregate in the *Col-0* × *Ull2–5* cross shown in Figure S10, yet there is no QTL at *CRY2* despite an association for JIC8W).

The *FLC* polymorphism identified by Caicedo et al. [33] showed a weak but consistent association with the response to differences in day length, SD/LD (V). A similar association was also obtained using mixed models. The *FLC* polymorphism was only segregating in one of our crosses, *Col-0* × *Edi-0*, the only one for which a strong QTL effect overlapping *FLC* was not seen (Figure 5). Note that this does not contradict a SD/LD (V) association since the phenotype measured in the cross was JIC4W.

*PHYC,* finally, was not significantly associated with any phenotype. However, in agreement with previous results [34], *PHYC* appeared to interact with latitude. The *PHYC* by Latitude interaction term was very significant for ±V (SD), and weakly significant for ±V (LD) and VERN. The latter two were also weakly significant in the mixed-model analyses. Although the *PHYC* polymorphism was segregating in all our crosses, we never observed a QTL overlapping it. Again, the phenotypes measured in the crosses are very different from the ones that appear to be associated with *PHYC*.

## Discussion

There is currently great interest in applying association mapping to a wide range of organisms, many of which (e.g., maize [12] and dog [35]) have heavily structured populations. Our study provides a dramatic example of the difficulties that may be encountered. Although our example may ultimately turn out to have been extreme, we would caution against premature optimism. Indeed, there should be no reason ever to *assume* anything about the importance of population structure in a particular study; it is straightforward to determine the distribution of test statistics empirically by calculating them across large numbers of loci. In genome-wide scans, large numbers of loci will by definition always be available, and for candidate locus studies, our results simply illustrate the need for an appropriate genomic control. Any association result not accompanied by a demonstration that the reported significance values are actually meaningful should be treated with suspicion (unless supported by strong experimental evidence).

We took advantage of our genome-wide data to evaluate published claims of association in A. thaliana. We found no evidence for association between the *CRY2* polymorphism reported by Olsen et al. [32] and any of our phenotypes, while the *FLC* polymorphism reported by Caicedo et al. [33] is at best weakly associated with the flowering response to differences in day length after vernalization. In contrast, the *PHYC* polymorphism reported by Balasubramanian et al. [34] does appear to show a significant interaction with latitude in determining vernalization response, in broad agreement with the original findings. While it is of course not possible to disprove a published association using a different sample and different phenotypic measurements, it is notable that neither Olsen et al. [32] nor Caicedo et al. [33] employed an appropriate genomic control, while Balasubramanian et al. [34] based their claim on a ranking of *p*-values (from a small genomic control) and, more importantly, several independent sources of evidence.

A number of statistical approaches for reducing confounding by population structure have been proposed. Methods, like “genomic control” [15], which simply rescale *p*-values without changing the rankings of loci, are not likely to be useful in genome-wide scans where the existence of true positives is not in doubt. Most biologists will simply rank the associations by nominal significance and proceed to test the most significant. Methods that attempt to separate true from spurious associations, like the ones used in this study, are of much greater interest. Our results demonstrate that such methods can be very effective at reducing (not eliminating) confounding. It is clear that this is a hard problem, and more theoretical work is certainly warranted. Comprehensive simulation studies (with known null distributions) would be required to determine how the different methods perform in terms of the false-positive rate. The mixed-model approach [14,36] seems particularly promising, and further research would probably be worthwhile. General principles for how kinship (the **K** matrix) should be estimated might be attainable, whereas the utility of estimators of population structure are likely to vary greatly from case to case. The underlying structure in our sample appears to be some form of isolation-by-distance [22], and both STRUCTURE [18] and PCA [17] appear to capture the relevant aspects of this structure reasonably well (Table 1). The first two principal components from the PCA are in fact strongly correlated with longitude and latitude (Figure S11).

At the same time, it is important to recognize that there are limits to what can be achieved using statistics. As noted above, any method that effectively removes confounding will also effectively remove true positives that are strongly correlated with population structure. Thus, while reducing confounding makes the common, widely distributed alleles of *FRI* stand out more clearly in our study, it also eliminates many of the best candidates for the extreme late-flowering phenotype characteristic of the northern accessions. Because these accessions are so distinct on a genome-wide level, any late-flowering allele shared by all or most of them would (and should) be removed by a statistical method that reduces confounding. There has to be segregation for mapping to work.

Interestingly, *FLC,* a central regulator of flowering, may be an example of this. Allelic variation at *FLC* has been shown to affect flowering [37,38], and five of our F2 crosses revealed a strong flowering QTL centered on it [28]. *FLC* expression is strongly correlated with flowering time (Table 1 and [39]). In our naive analysis (i.e., without correcting for population structure), *FLC* is one of the strongest associations for many phenotypes; however, because the significance is due to allele sharing among a large subset of northern accessions, it is down-weighted when population structure is taken into account (this can be seen in Figure 5, for example). It should be noted that the strong association we find at *FLC* (whether causative or spurious) does not correspond to the association previously reported by Caicedo et al. [33]. As discussed above, the polymorphism studied by these authors is also associated with several of our phenotypes (Tables S2–S4), but the associations are much weaker.

In conclusion, we should emphasize that although this paper has focused on the difficulties caused by confounding, we do not mean to say that these studies are impossible, merely that caution is needed. The statistical methods we used look promising, and we did identify several interesting associations. It must be remembered that, in a small and heavily structured sample such as the one used here, we should really only expect to be able to find polymorphisms with a major effect on the phenotypic variation that segregate widely across the geographic distribution of the sample. *FRI* is an example, and we do indeed find it. Of the new associations we have identified (Table 4), the most promising is probably the common polymorphism at *CLF,* which may be a major factor in determining the differential flowering response to day length. However, the large fraction of the phenotypic variation that is due to the very late accessions probably cannot be dissected further using this sample. There is every reason to believe that genome-wide association studies involving adequate marker densities and larger samples will be very fruitful.

## Materials and Methods

### Plant materials.

The accessions used have been described in previous papers [13,22].

### Genotypes.

We used the resequencing data described in reference [13], plus approximately 100 fragments in and around several flowering time–related genes: *FLM* [40,41], *FVE* [42], *FPA* [43], *VRN1* [44], *FRI* [21], *LD* [45], *FCA* [46], *VRN2* [47], *FLC* [19,20], and *FY* [48]. Note that since the accessions are inbred, these data are in the form of short haplotypes. Furthermore, linkage disequilibrium is extensive, and there was no evidence of recombination in about three quarters of the fragments [22]. Given this, some association analyses were carried out treating the fragments as multi-allelic loci, with each haplotype corresponding to an allele. To avoid too many rare haplotypes, all singleton polymorphisms were ignored. Remaining rare haplotypes (those with frequency less than 5%) were pooled.

The *CRY2* haplogroups of Olsen et al. [32] were typed in our sample by resequencing two fragments in the *CRY2* gene. Similarly, the *FLC* haplotypes of Caicedo et al. [33] were identified based on their SNP 2553 and our resequencing data. The *PHYC* genotyping was done by S. Balasubramanian in D. Weigel's laboratory using published methods [34].

### Phenotypes.

Plants were grown under either long-day (16 h light/8 h dark) or short-day (8 h light/16 h dark) conditions, using a randomized design (except with respect to growth chambers). Seeds were sown on soil and stratified for 2–3 d at 4 °C to synchronize germination. At USC, experiments were carried out in Conviron MTPS72 growth chambers programmed to have a gradual transition between light and dark conditions. Plants were grown at a constant temperature of 18 °C. The vernalization treatment was done at 4 °C on 3–4-d-old seedlings, using the same light conditions as for the unvernalized plants. Conditions at JIC were similar, but plants were grown in a custom-built walk-in controlled environment room using a temperature of 23 °C, and vernalization was done at 5 °C under short-day (8h light) conditions in a Sanyo-Gallenkamp CE with cool white fluorescent lamps only. There were thus clear differences in both temperature and light quality between the USC and JIC experiments.

Flowering times were measured in days from germination to the first opening of flowers (USC) or to plant height reached at 5 cm (JIC). All measurements were replicated (using six plants at USC and ten plants at JIC). The variance across replicates was trivial compared to the variance across accessions; including it in the analyses did not seem to affect results and we therefore carried out all analyses using the accession means. The experiments were continued until either all individuals had flowered or the rate of new individuals starting to flower was deemed to have reached zero. When an experiment was stopped before all accessions had flowered, the remaining individuals were assigned the stopping date as phenotype (the resulting phenotypic distributions can be seen in Figure S1). The precise value of the phenotype assigned to these nonflowering accessions did not seem to affect the analyses (unpublished data).

As indicated in Table 1, we also considered several ratios of phenotypes in order to look for responses to particular treatments. The rate of response to vernalization was studied using the 0-, 2-, 4-, and 8-wk vernalization treatments from JIC. We did this by first an exponential decay model, *FT*(*t*) *= ae^−bt^*, where *FT*(*t*) is the flowering time after *t* weeks of vernalization. The decay slope *b* was then used as the phenotype in association mapping.

*FRI* and *FLC* expression was measured as described in Shindo et al. [29]. All phenotypes were normalized before analysis.

### Association mapping.

Our basic analyses, including our implementation of “structured association” and estimation of population structure using STRUCTURE [18] have been described elsewhere [13,22]. Our mixed-model approach follows that of Yu et al. [14] closely. The vector of phenotypes, **y**, is modeled as

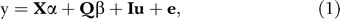
where **X** contains the genotypes, *α* is vector of allele effects to be estimated, **Q** contains the population assignments by STRUCTURE, *β* is vector of subpopulation effects, **I** is an identity matrix, **u** are the random deviates due to genome-wide relatedness, and **e** are random deviates due to noise. Crucially, the phenotypic covariance matrix is assumed to have the form

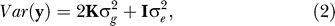
where **K** is the matrix of kinship coefficients, 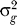 is the genetic variance attributable to genome-wide effects, and 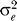 is the residual variance. In addition to the full model described by Equation 1, we considered models without the population structure term, and without the kinship term (this is “structured association”).

We also varied how we estimated the kinship coefficients. Results are presented for two different choices: the **K** used by Yu et al. [14], and our own K*. The former contains the relative kinship coefficients of Ritland [24] estimated using SPAGeDi [49] from about 12,000 non-singleton SNPs identified by Nordborg et al. [22]. In this approach, the kinship coefficient between individuals *i* and *j* is defined as *K_ij_ =* (*Q_ij_ − Q_m_*)/(1 − *Q_m_*), where *Q_ij_* is the probability of identity in state for random loci from *i* and *j*, and *Q_m_* is the average probability of identity by state for loci from random individuals from the sample. With this definition, a negative relative kinship coefficient means that the particular pair of accessions is less related than random individuals. In the analysis, all negative values were set to zero [14].

As an alternative to this approach, we introduced K*, which contains kinship coefficients defined simply as the proportion of shared haplotypes (with singletons removed) for each pair of individuals.

All analyses were done using standard methods as implemented in SAS using PROC MIXED. The significance of the fixed effects was obtained from F-tests with the denominator degrees of freedom obtained using the Satterthwaite method .

We tested the principal components method using a modified version of EIGENSTRAT [17]. Since our phenotypes are quantitative rather than binary, we replaced the χ^2^ statistic with Spearman's rank correlation.

Finally, we tried a mixed-model approach where we simply replaced the **Q** matrix produced by STRUCTURE with a **P** matrix containing the eight top principal components from EIGENSTRAT.

### Data perturbation simulations.

A simulation scheme similar to that of Yu et al. [14] was used. The idea was to perturb the existing phenotypes (we used LDV) by adding a constant additive effect to a randomly chosen causal SNP, thus maintaining the complicated correlation structure of the data.

Causal SNPs were randomly chosen from SNPs with minor allele frequency in the 20%–40% range. A fixed genetic effect was added to each causal SNP. Fixed effects ranging from 0.2 to 1.4 times the standard deviation of the phenotype were used. The percentage of the total phenotypic variation explained by each causal SNP (which depends on the frequency of the SNP as well as its effect) was calculated using a standard linear regression of phenotype on SNP.

### Linkage mapping in F2 crosses.

QTL mapping for flowering time was carried out in six F2 populations generated by crossing *Col-0* to six different accessions chosen because of their differential response in our association study. Four of these crosses have previously reported by Shindo et al. [28], whereas two *(Col-0* × *Knox-18* and *Col-0* × *RRS-10)* are reported for the first time here. For details about the crosses, please see [28].

Analysis was done using composite interval mapping as implemented in QTL Cartographer [50].

## Supporting Information

Dataset S1 Phenotypes Used in the Analysis (5 KB CSV)

Dataset S2 All Sequence Alignments Not Previously Published [13], Plus Tables of Haplotype and SNP Genotypes Used in the Analyses (1.0 MB ZIP)

Dataset S3 Output of Genome-Wide Scan for All Methods and Phenotypes (8.0 MB ZIP)

Figure S1 Histograms Showing the Distributions of the Various Phenotypes (10 KB PDF)

Figure S2 Cumulative Distribution of *p*-Values from Haplotype-Based Tests (1.2 MB PDF)

Figure S3 Power Comparison for Haplotype-Based Tests (86 KB PDF)

Figure S4 Genome-Wide Association Scans Using the K* Model Peak heights represent negative log *p*-values from tests of association between genotype and phenotype. The dashed grey line marks a nominal significance level of 0.1%. The position of the candidate genes are highlighted. Nominally significant associations occur throughout the genome, and often correlated across phenotypes.
(132 KB PDF)

Figure S5 Genome-Wide Association Scans Using the **Q** + **K** Model (131 KB PDF)

Figure S6 Genome-Wide Association Scans Using the **Q** + K* Model (133 KB PDF)

Figure S7 Genome-Wide Association Scans Using the **P** + **K** Model (126 KB PDF)

Figure S8 Genome-Wide Association Scans Using the **P** + K* Model (131 KB PDF)

Figure S9 Comparison of Association and Linkage Mapping Results for the Phenotype JIC0W The position of certain candidate genes are highlighted in yellow.
(A) The QTL LOD scores for the cross *Col-0* × *Kno-18* (blue curve). The dashed grey line marks a genome-wide permutation significance level of 5%.
(B ) Negative log *p*-values from a genome-wide scan using the **Q** + **K** model.
(C) Negative log *p*-values from a genome-wide scan using a naive Kruskal-Wallis approach.
(B and C) The dashed grey line marks a nominal significance level of 0.1%, and the colored stars denote associations that should correspond to QTL in the cross.
(64 KB PDF)

Figure S10 Comparison of Association and Linkage Mapping Results for the Phenotype JIC8W The position of certain candidate genes are highlighted in yellow.
(A) The QTL LOD scores for three crosses: *Col-0* × *RRS-10* (black curve), *Col-0* × *Var2–6* (red curve), and *Col-0* × *Ull2–5* (green curve). The dashed grey line marks a genome-wide permutation significance level of 5%.
(B) Negative log *p*-values from a genome-wide scan using the **Q** + **K** model.
(C) Negative log *p*-values from a genome-wide scan using a naive Kruskal-Wallis approach.
In (B) and (C), the dashed grey line marks a nominal significance level of 0.001, and the colored stars denote associations that should correspond to QTL in the cross displayed using the same color.
(71 KB PDF)

Figure S11 The Top Two Principal Components from EIGENSTRAT Superimposed on Geographic Origins (54 KB PDF)

Figure S12 Cumulative Densities of *p*-Values for Association between the AFLP Markers and Phenotypes Used by Olsen et al. [32] and Caicedo et al. [33] (11 KB PDF)

Table S1 Mixed-Model Analysis of Previously Published Candidate Polymorphisms in *CRY2* [32], *FLC* [33], and *PHYC* [34] (17 KB PDF)

Table S2 Basic Linear Model Analysis of Previously Published Candidate Polymorphisms in *CRY2* [32], *FLC* [33], and *PHYC* [34] (25 KB PDF)

Table S3 Linear Model Analysis (Including a Locus by Latitude Interaction Term) of Previously Published Candidate Polymorphisms in *CRY2* [32], *FLC* [33], and *PHYC* [34] (18 KB PDF)

Table S4 Linear Model Analysis (Including Both Locus by Latitude and Locus by *FRI* Interaction Terms) of Previously Published Candidate Polymorphisms in *CRY2* [32], *FLC* [33], and *PHYC* [34] (18 KB PDF)

## Abbreviations

JIC: John Innes Centre
PCA: principal components analysis
QTL: quantitative trait locus
QTN: quantitative trait nucleotide
SNP: single nucleotide polymorphism
USC: University of Southern California
