# Supplementary material for: An Arabidopsis Example of Association Mapping in Structured Samples
Source: PLoS Genet. 2007 Jan 19;3(1):e4. doi: 10.1371/journal.pgen.0030004 (PMC1779303; doi:10.1371/journal.pgen.0030004)
Supplement: Supplementary file 2 [file pgen.0030004.sg002.pdf]

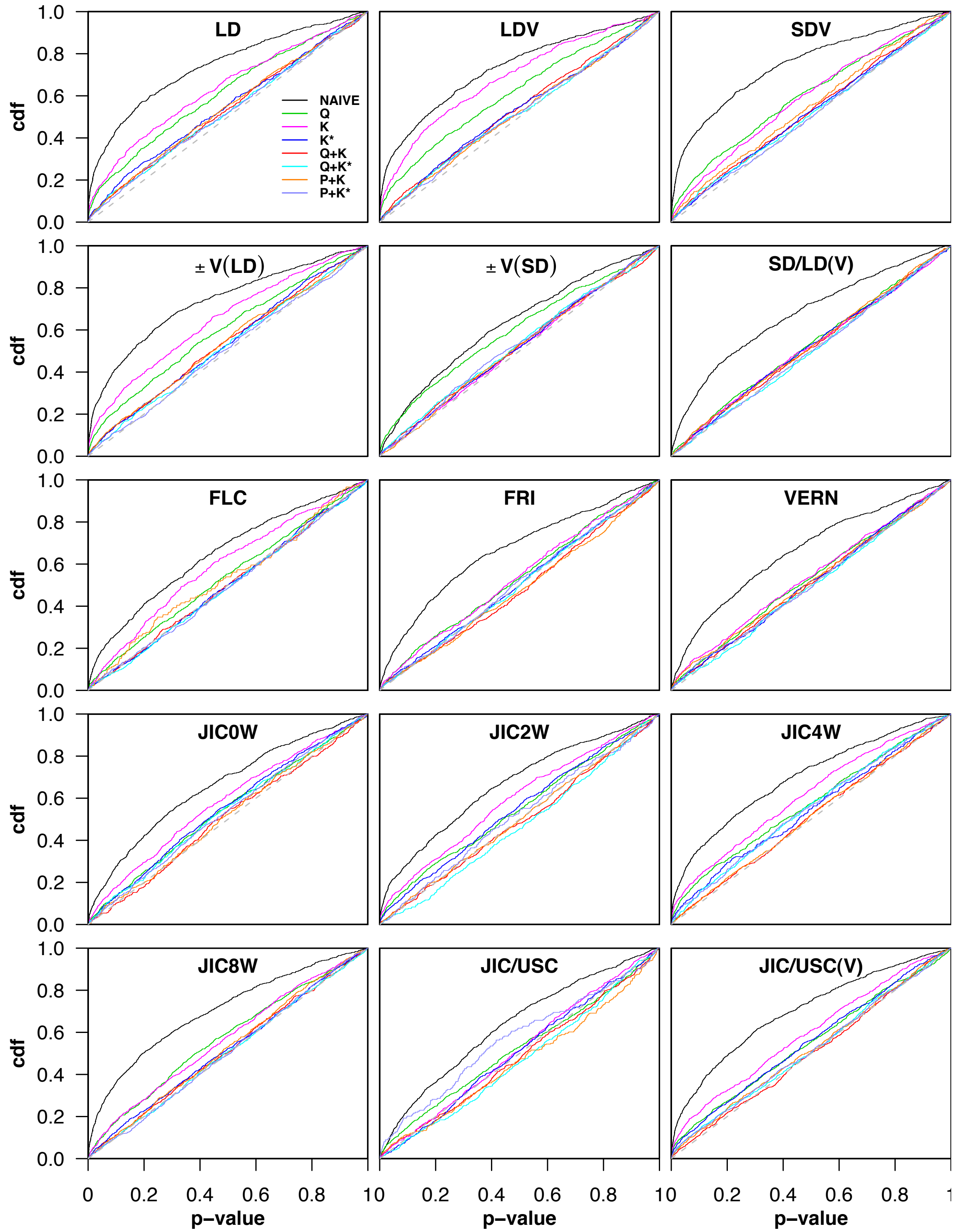

Figure S2: The cumulative distribution of p-values in genome-wide scans for all phenotypes. The different curves correspond to different approaches for correcting for population structure (described in Table 2). Without correction for population structure, all p-value distributions are strongly skewed towards significance. These results are for associations between phenotype and haplotypes (cf. Figure 2).
