# Supplementary material for: An Arabidopsis Example of Association Mapping in Structured Samples
Source: PLoS Genet. 2007 Jan 19;3(1):e4. doi: 10.1371/journal.pgen.0030004 (PMC1779303; doi:10.1371/journal.pgen.0030004)
Supplement: Supplementary file 3 [file pgen.0030004.sg003.pdf]

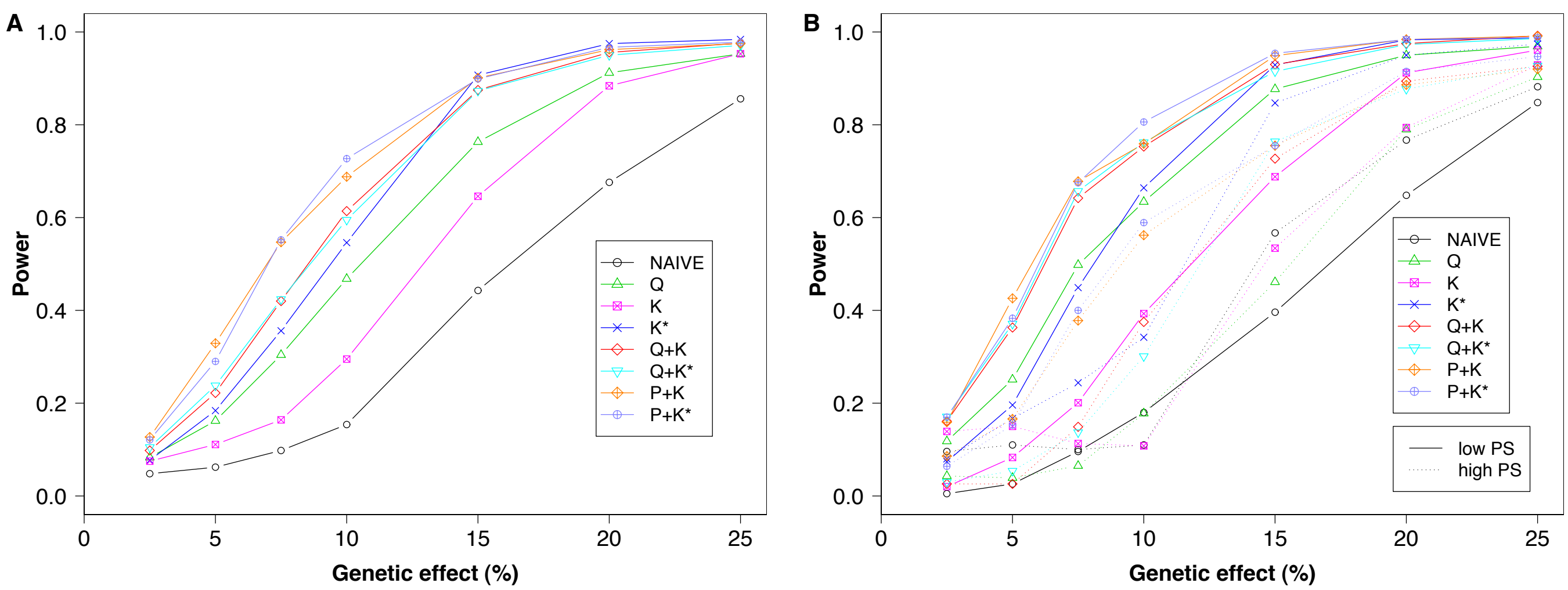

Figure S3: Power of the different methods to detect QTN using a 5% significance level, calculated as in Figure 3 but using haplotype markers. (A) Power calculated across all simulations. (B) Power calculated separately for QTN that showed strong and weak correlation with population structure.
