# Supplementary material for: An Arabidopsis Example of Association Mapping in Structured Samples
Source: PLoS Genet. 2007 Jan 19;3(1):e4. doi: 10.1371/journal.pgen.0030004 (PMC1779303; doi:10.1371/journal.pgen.0030004)
Supplement: Supplementary file 4 [file pgen.0030004.sg004.pdf]

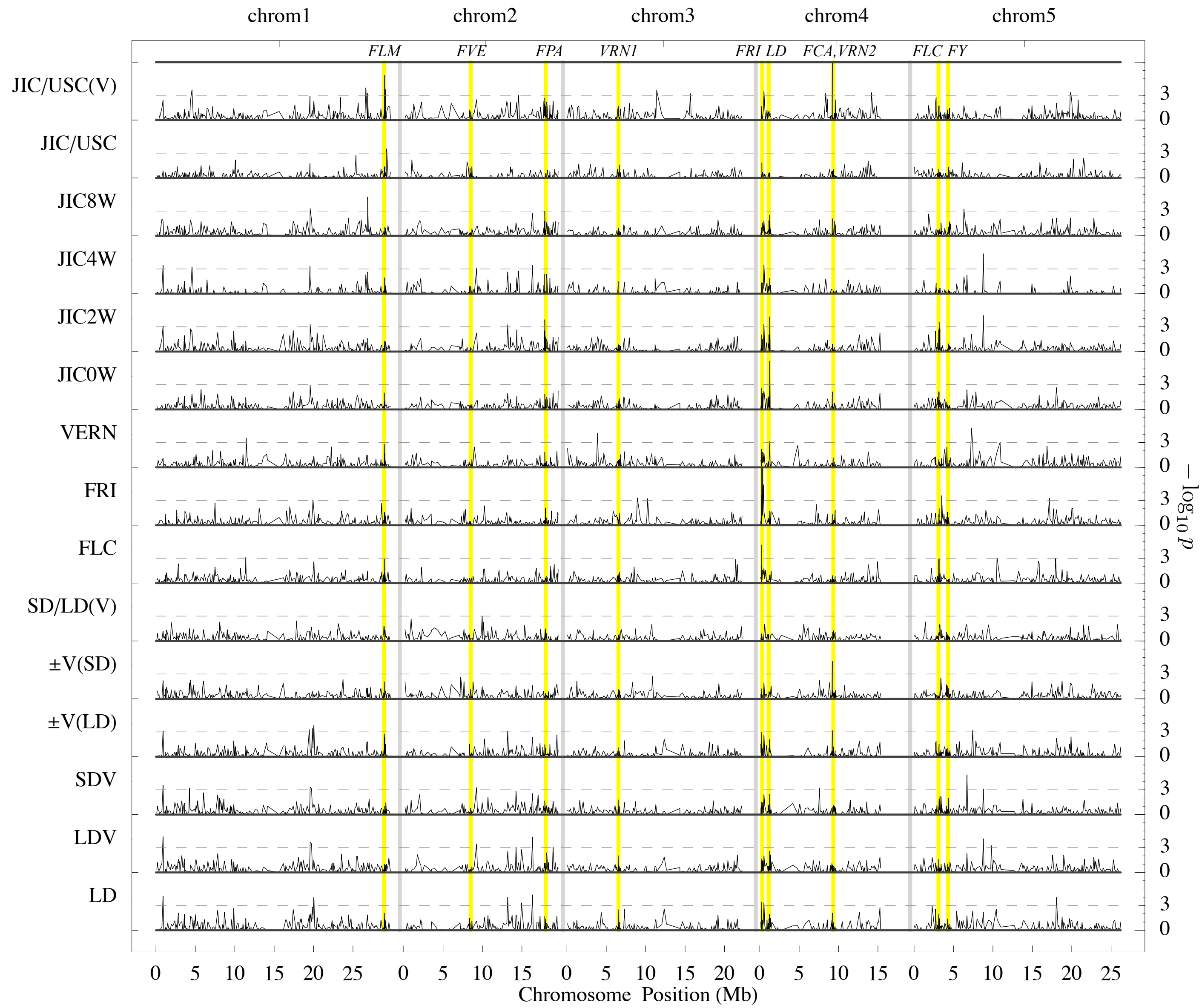

Figure S4: Genome-wide association scans using the $\mathbf{K}^\star$ model. Peak heights represent negative log p-values from tests of association between genotype and phenotype. The dashed grey line marks a nominal significance level of 0.1%. The position of the candidate genes are highlighted. Nominally significant associations occur throughout the genome, and often correlated across phenotypes.
