# Supplementary material for: An Arabidopsis Example of Association Mapping in Structured Samples
Source: PLoS Genet. 2007 Jan 19;3(1):e4. doi: 10.1371/journal.pgen.0030004 (PMC1779303; doi:10.1371/journal.pgen.0030004)
Supplement: Supplementary file 6 [file pgen.0030004.sg006.pdf]

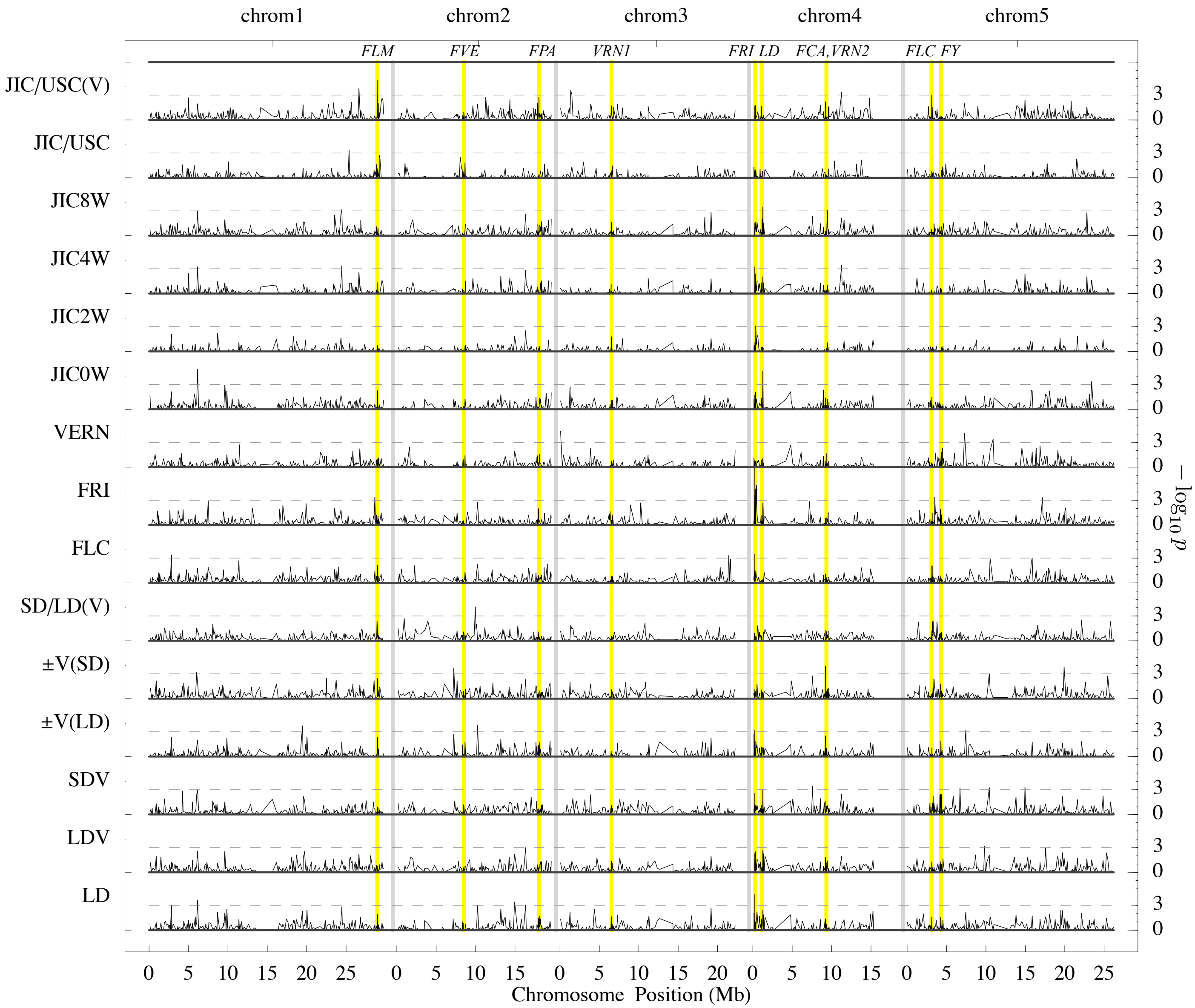

Figure S6: Same as Figure S4, but for the $\mathbf{Q} + \mathbf{K}^{\star}$ model.
