# Supplementary material for: An Arabidopsis Example of Association Mapping in Structured Samples
Source: PLoS Genet. 2007 Jan 19;3(1):e4. doi: 10.1371/journal.pgen.0030004 (PMC1779303; doi:10.1371/journal.pgen.0030004)
Supplement: Supplementary file 9 [file pgen.0030004.sg009.pdf]

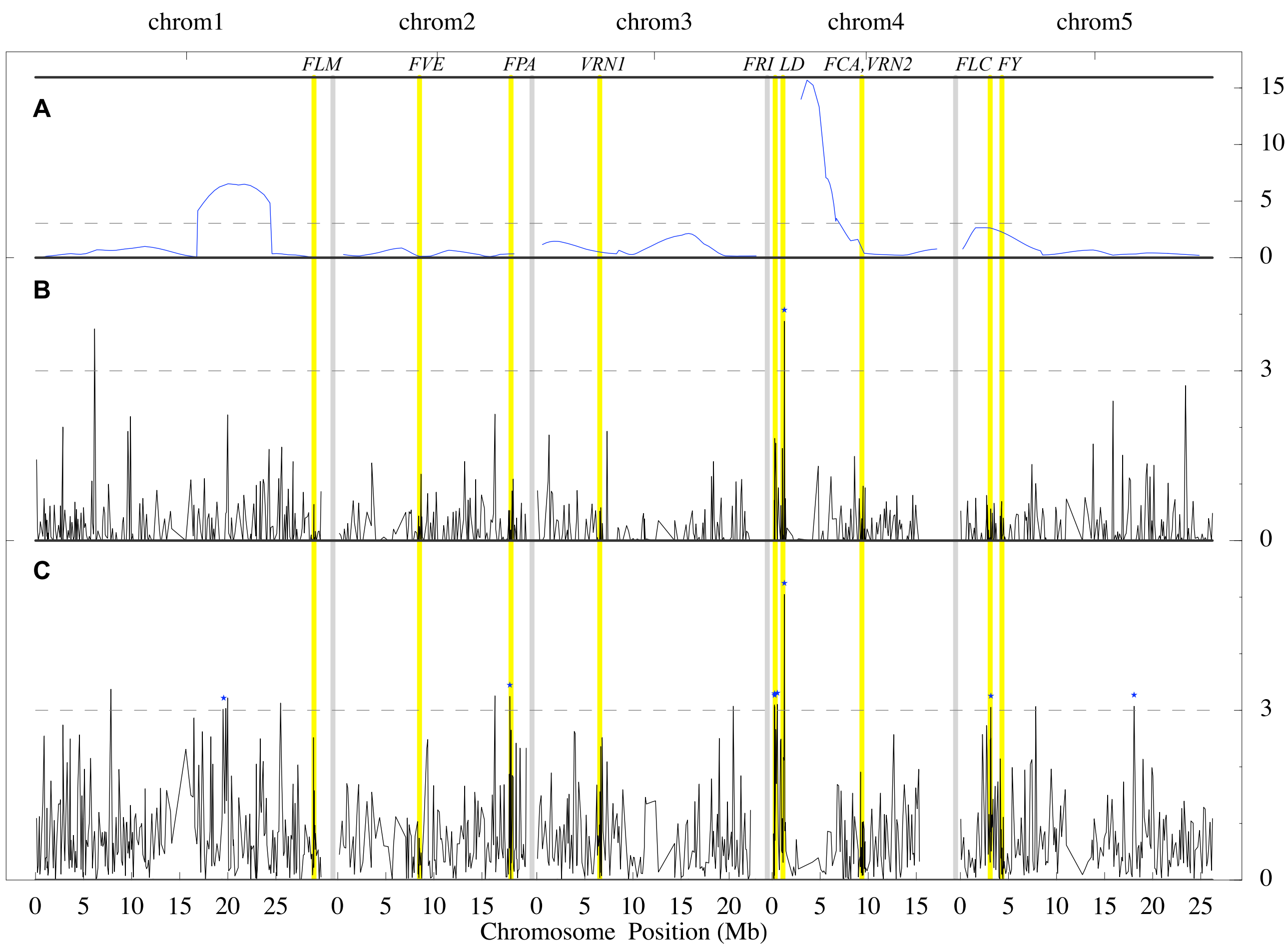

Figure S9: Comparison of association and linkage mapping results for the phenotype JIC0W. The position of certain candidate genes are highlighted in yellow. (A) The QTL LOD scores for the cross *Col-0* × *Kno-18* (blue curve). The dashed grey line marks a genome-wide permutation significance level of 5%. (B) Negative log p-values from a genome-wide scan using the $\mathbf{Q} + \mathbf{K}$ model. (C) Negative log p-values from a genome-wide scan using a naive Kruskal-Wallis approach. In (B) and (C), the dashed grey line marks a nominal significance level of 0.1%, and the colored stars denote associations that should correspond to QTL in the cross.
