# Supplementary material for: An Arabidopsis Example of Association Mapping in Structured Samples
Source: PLoS Genet. 2007 Jan 19;3(1):e4. doi: 10.1371/journal.pgen.0030004 (PMC1779303; doi:10.1371/journal.pgen.0030004)
Supplement: Supplementary file 10 [file pgen.0030004.sg010.pdf]

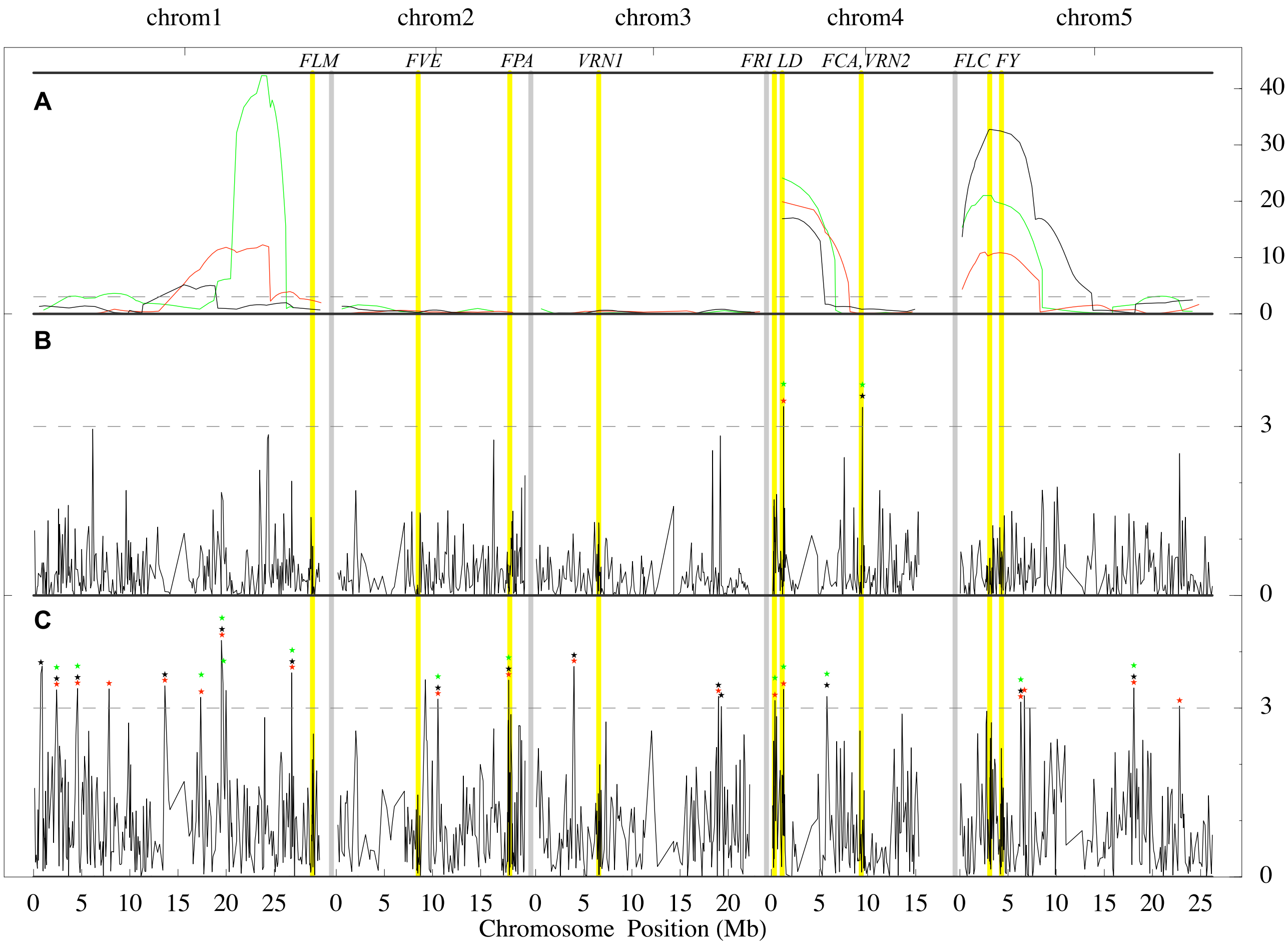

Figure S10: Comparison of association and linkage mapping results for the phenotype JIC8W. The position of certain candidate genes are highlighted in yellow. (A) The QTL LOD scores for three crosses: *Col-0* $\times$ *RRS-10* (black curve), *Col-0* $\times$ *Var2-6* (red curve) and *Col-0* $\times$ *Ull2-5* (green curve). The dashed grey line marks a genome-wide permutation significance level of 5%. (B) Negative log p-values from a genome-wide scan using the $\mathbf{Q}+\mathbf{K}$ model. (C) Negative log p-values from a genome-wide scan using a naive Kruskal-Wallis approach. In (B) and (C), the dashed grey line marks a nominal significance level of 0.001, and the colored stars denote associations that should correspond to QTL in the cross displayed using the same color
