# Supplementary material for: An Arabidopsis Example of Association Mapping in Structured Samples
Source: PLoS Genet. 2007 Jan 19;3(1):e4. doi: 10.1371/journal.pgen.0030004 (PMC1779303; doi:10.1371/journal.pgen.0030004)
Supplement: Supplementary file 11 [file pgen.0030004.sg011.pdf]

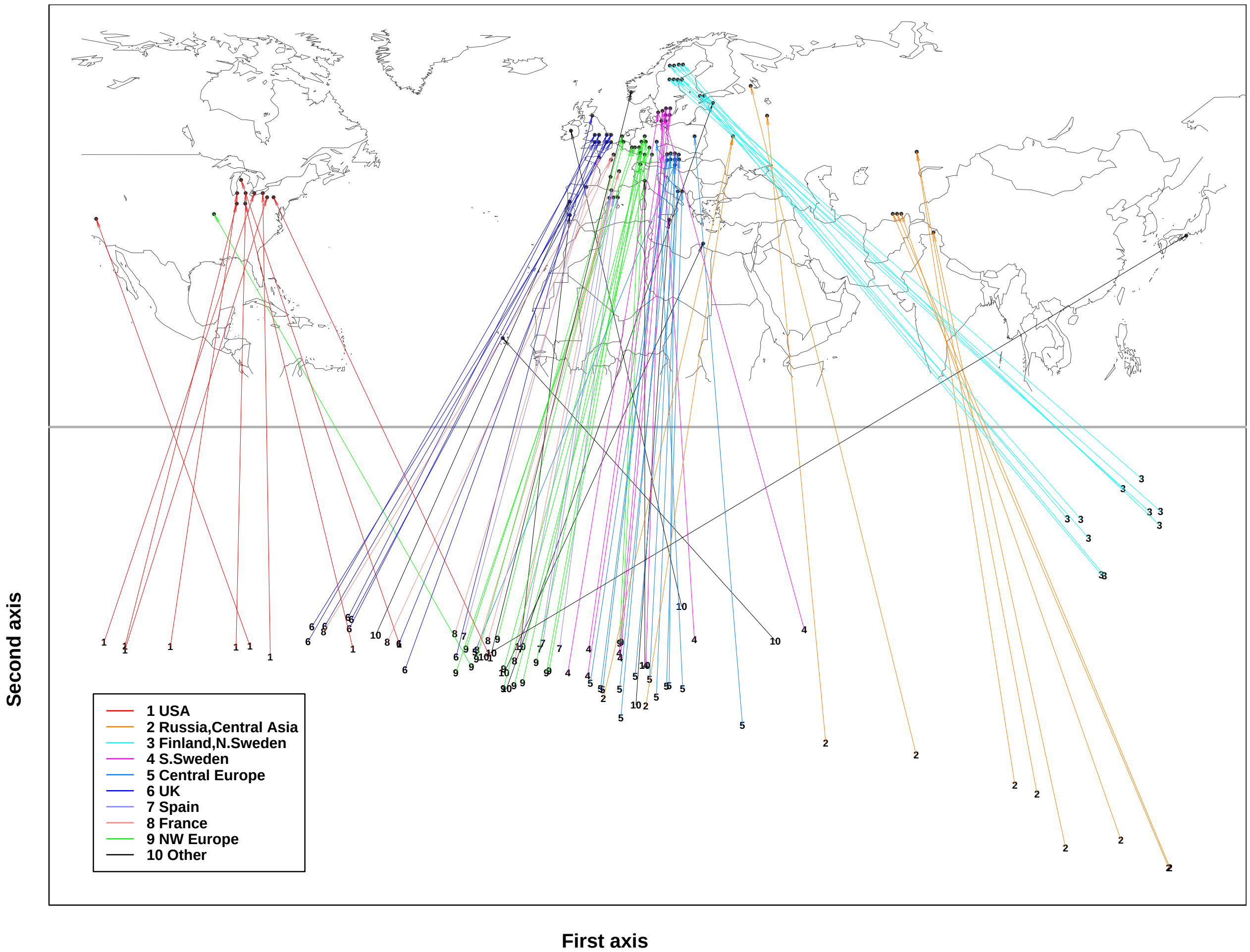

Figure S11: The top two principal components of genetic variation from `EIGENSTRAT` [17]. The lower panel shows the projected values along the top two axes, and the upper panel shows the corresponding geographic origin of each accession.
