# Supplementary material for: An Arabidopsis Example of Association Mapping in Structured Samples
Source: PLoS Genet. 2007 Jan 19;3(1):e4. doi: 10.1371/journal.pgen.0030004 (PMC1779303; doi:10.1371/journal.pgen.0030004)
Supplement: Supplementary file 12 [file pgen.0030004.sg012.pdf]

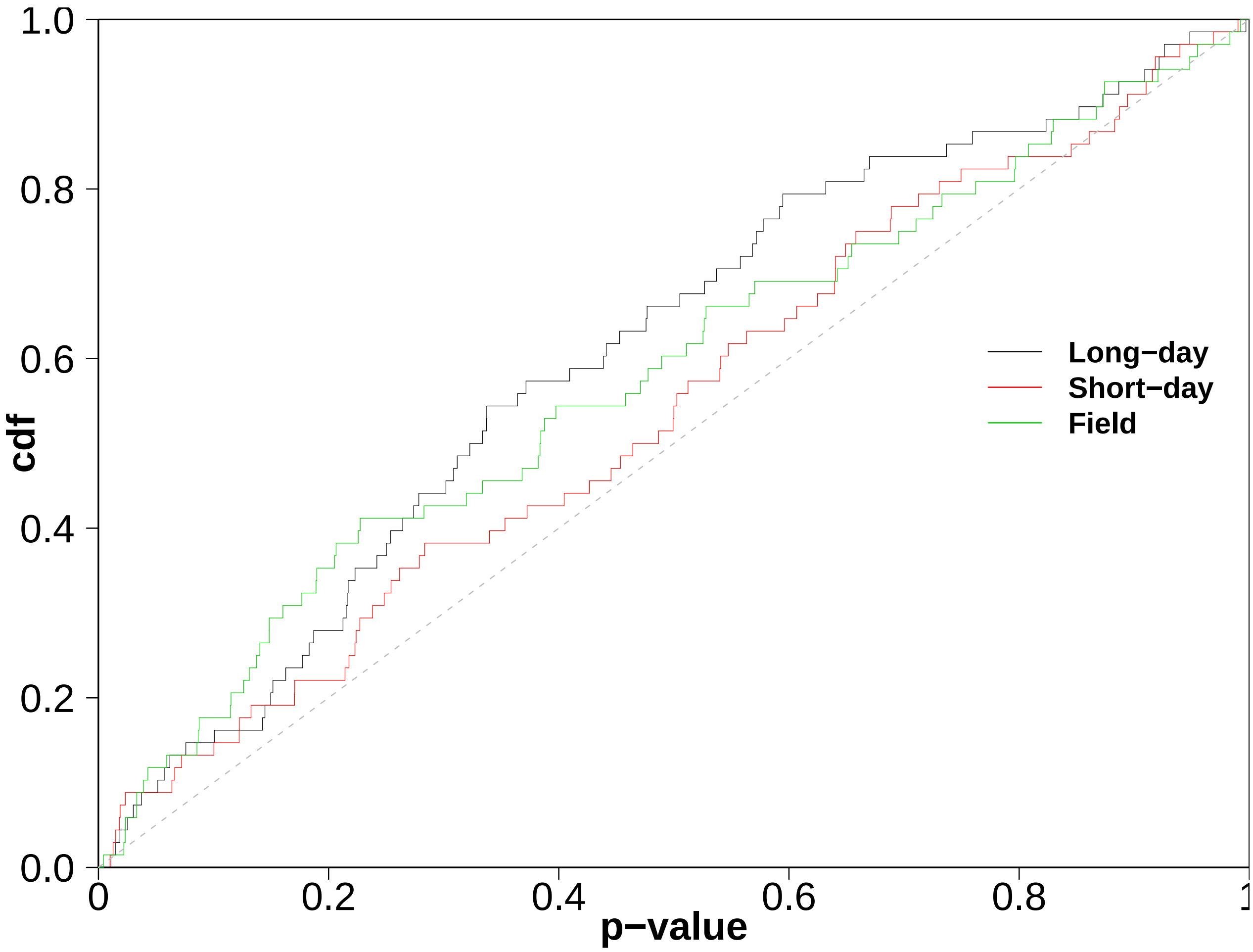

Figure S12: The cumulative distribution of p-values from naive tests of association between the 79 AFLP markers and phenotypes used by Olsen et al. [32] and Caicedo et al. [33]. Note that the p-values reported in these papers refer to tag-SNPs that distinguish major haplotypes, and thus cannot be directly compared with this distribution. See text for details.
