# Supplementary material for: An Arabidopsis Example of Association Mapping in Structured Samples
Source: PLoS Genet. 2007 Jan 19;3(1):e4. doi: 10.1371/journal.pgen.0030004 (PMC1779303; doi:10.1371/journal.pgen.0030004)
Supplement: Supplementary file 13 [file pgen.0030004.st001.pdf]

Table S1. Mixed-model analysis of candidate polymorphisms.

Phenotype	$\mathbf{K}^\star$	$\mathbf{Q}+\mathbf{K}$	$\mathbf{Q}+\mathbf{K}^\star$	$\mathbf{P}+\mathbf{K}$	$\mathbf{P}+\mathbf{K}^\star$
			CRY2 [32]		
LD	0.35	0.10	0.19	0.26	0.28
LDV	0.25	0.13	0.22	0.30	0.41
SD	0.25	0.15	0.14	NA	0.34
SDV	0.20	0.33	0.33	NA	0.42
JIC0W	0.44	NA	0.77	0.69	0.67
JIC2W	0.15	0.29	0.24	0.36	NA
JIC4W	NA	0.33	NA	0.48	NA
JIC8W	0.17	0.20	0.31	0.22	0.33
±V (LD)	NA	0.21	0.30	0.34	0.39
±V (SD)	0.44	0.61	0.59	0.52	0.54
SD/LD (V)	0.44	0.72	0.70	0.56	0.54
VERN	0.58	0.32	0.34	0.34	0.37
JIC/USC	0.26	NA	NA	0.55	0.88
JIC/USC (V)	0.72	0.89	0.83	NA	0.94
FRI	0.24	0.28	0.25	0.23	0.21
FLC	0.58	0.64	0.52	NA	0.50
			FLC [33]		
LD	0.44	0.48	0.65	0.27	0.47
LDV	0.94	0.89	0.95	0.96	1.00
SD	0.06	NA	0.10	NA	**0.04**
SDV	0.11	0.06	0.08	NA	0.09
JIC0W	0.18	NA	0.36	0.18	0.22
JIC2W	0.11	0.23	0.23	0.15	NA
JIC4W	0.17	0.38	NA	0.34	NA
JIC8W	0.19	0.29	0.24	0.12	0.11
±V (LD)	0.29	0.47	0.55	0.28	0.39
±V (SD)	0.95	0.57	0.71	0.88	0.75
SD/LD (V)	**0.05**	**0.03**	**0.04**	0.10	0.12
VERN	0.37	0.58	0.60	0.44	0.56
JIC/USC	0.08	0.08	0.12	NA	NA
JIC/USC (V)	0.11	0.25	0.41	0.26	0.42
FRI	0.17	0.51	0.51	NA	0.39
FLC	**0.05**	0.12	0.13	NA	0.09
			PHYC [34]		
LD	0.14	0.21	0.20	0.21	0.23
LDV	0.92	0.43	0.47	0.55	0.64
SD	0.16	0.25	0.22	NA	0.20
SDV	0.19	0.45	0.46	NA	0.39
JIC0W	0.36	0.18	0.22	0.21	0.22
JIC2W	0.27	0.26	0.37	0.26	NA
JIC4W	0.70	0.73	NA	0.71	NA
JIC8W	0.64	NA	0.34	0.45	0.48
±V (LD)	0.07	**0.04**	0.06	0.07	0.07
±V (SD)	0.92	0.66	0.58	0.92	0.95
SD/LD (V)	0.08	0.13	0.15	0.19	0.22
VERN	0.07	**0.01**	NA	0.06	0.07
JIC/USC	0.83	0.61	0.55	0.57	NA
JIC/USC (V)	0.94	0.97	0.92	NA	0.59
FRI	0.90	0.96	0.96	0.97	0.99
FLC	0.43	0.13	0.19	NA	0.31
