# Supplementary material for: An Arabidopsis Example of Association Mapping in Structured Samples
Source: PLoS Genet. 2007 Jan 19;3(1):e4. doi: 10.1371/journal.pgen.0030004 (PMC1779303; doi:10.1371/journal.pgen.0030004)
Supplement: Supplementary file 14 [file pgen.0030004.st002.pdf]

Table S2. Linear model analysis of candidate polymorphisms with and without cofactors.

	X^a					X^b		
**Phenotype**	**raw**[c]	**%SNP**[d]	**%HAP**[e]	**Latitude**	**FRI**	**raw**[c]	**%SNP**[d]	**%HAP**[e]
				$X = CRY2$ [32]				
LD	0.11	(0.35)	(0.54)	**2E-07**	**6E-07**	**4E-04**	(0.11)	(0.29)
LDV	**0.03**	(0.19)	(0.36)	**1E-06**	**0.01**	**1E-03**	(0.09)	(0.23)
SD	0.11	(0.32)	(0.51)	**3E-04**	**8E-05**	**3E-03**	(0.11)	(0.28)
SDV	**0.01**	(0.07)	(0.16)	**3E-06**	0.13	**6E-04**	(**0.04**)	(0.12)
JIC0W	0.25	(0.40)	(0.51)	0.07	**1E-05**	**3E-03**	(0.05)	(0.12)
JIC2W	0.11	(0.28)	(0.41)	**4E-04**	**1E-05**	**6E-04**	(0.07)	(0.17)
JIC4W	0.07	(0.24)	(0.41)	**8E-05**	**7E-04**	**8E-04**	(0.07)	(0.21)
JIC8W	**0.03**	(0.12)	(0.22)	0.17	**0.02**	**1E-03**	(**0.03**)	(0.08)
±V (LD)	0.31	(0.54)	(0.71)	**3E-05**	**5E-07**	**0.01**	(0.18)	(0.37)
±V (SD)	0.22	(0.32)	(0.40)	**0.03**	**3E-03**	0.37	(0.50)	(0.59)
SD/LD (V)	0.08	(0.18)	(0.25)	0.07	0.44	0.12	(0.23)	(0.34)
VERN	0.61	(0.72)	(0.80)	0.09	**3E-05**	0.44	(0.63)	(0.75)
JIC/USC	0.18	(0.25)	(0.31)	**0.02**	0.61	0.14	(0.22)	(0.29)
JIC/USC (V)	0.18	(0.36)	(0.52)	**8E-03**	**2E-03**	**0.02**	(0.13)	(0.30)
FRI	0.10	(0.19)	(0.27)	0.21	**1E-11**	**2E-03**	(0.06)	(0.12)
FLC	0.73	(0.80)	(0.85)	0.90	**9E-09**	0.73	(0.84)	(0.90)
				$X = FLC$ [33]				
LD	0.22	(0.50)	(0.65)	**1E-08**	**2E-06**	0.52	(0.79)	(0.91)
LDV	0.65	(0.79)	(0.88)	**1E-07**	**0.01**	0.98	(0.99)	(1.00)
SD	**0.03**	(0.18)	(0.33)	**1E-05**	**1E-04**	0.07	(0.35)	(0.56)
SDV	**0.03**	(0.14)	(0.24)	**5E-08**	0.16	**0.02**	(0.17)	(0.33)
JIC0W	0.10	(0.21)	(0.31)	**0.01**	**3E-05**	0.21	(0.42)	(0.61)
JIC2W	0.05	(0.19)	(0.33)	**3E-05**	**3E-05**	0.17	(0.47)	(0.64)
JIC4W	0.06	(0.22)	(0.38)	**5E-06**	**8E-04**	0.12	(0.41)	(0.62)
JIC8W	0.07	(0.21)	(0.34)	0.05	**0.01**	0.17	(0.36)	(0.51)
±V (LD)	0.19	(0.42)	(0.59)	**2E-06**	**2E-06**	0.49	(0.74)	(0.87)
±V (SD)	0.73	(0.78)	(0.83)	**0.01**	**2E-03**	0.36	(0.50)	(0.59)
SD/LD (V)	**3E-03**	(**0.02**)	(**0.03**)	**0.01**	0.27	**1E-03**	(**0.01**)	(**0.02**)
VERN	0.10	(0.23)	(0.34)	**0.05**	**6E-05**	0.29	(0.49)	(0.66)
JIC/USC	**0.02**	(**0.05**)	(0.06)	**0.03**	0.58	**0.02**	(0.05)	(0.07)
JIC/USC (V)	**0.01**	(0.10)	(0.25)	**1E-03**	**2E-03**	**0.04**	(0.19)	(0.39)
FRI	0.07	(0.15)	(0.23)	**0.05**	**4E-10**	0.35	(0.54)	(0.71)
FLC	**0.03**	(0.09)	(0.13)	0.69	**2E-08**	0.16	(0.37)	(0.54)
				$X = PHYC$ [34]				
LD	0.10	(0.33)	(0.53)	**1E-07**	**1E-06**	**0.01**	(0.26)	(0.51)
LDV	0.68	(0.81)	(0.89)	**7E-08**	**0.01**	0.49	(0.74)	(0.87)
SD	0.21	(0.46)	(0.63)	**1E-04**	**1E-04**	0.12	(0.43)	(0.64)
SDV	0.13	(0.31)	(0.46)	**1E-06**	0.14	0.07	(0.29)	(0.49)
JIC0W	0.39	(0.54)	(0.66)	**0.02**	**3E-05**	0.16	(0.37)	(0.54)
JIC2W	0.26	(0.46)	(0.57)	**1E-04**	**3E-05**	0.07	(0.32)	(0.50)
JIC4W	0.60	(0.78)	(0.88)	**7E-06**	**1E-03**	0.36	(0.68)	(0.82)
JIC8W	0.82	(0.88)	(0.94)	**0.04**	**0.02**	0.94	(0.97)	(0.98)
±V (LD)	0.03	(0.17)	(0.32)	**4E-05**	**5E-07**	**2E-03**	(0.13)	(0.30)
±V (SD)	0.68	(0.75)	(0.80)	**0.02**	**3E-03**	0.68	(0.76)	(0.83)
SD/LD (V)	0.08	(0.17)	(0.24)	0.08	0.44	0.09	(0.20)	(0.29)
VERN	0.06	(0.15)	(0.26)	0.16	**2E-05**	**0.02**	(0.12)	(0.23)
JIC/USC	0.69	(0.72)	(0.79)	**0.03**	0.62	0.68	(0.71)	(0.79)
JIC/USC (V)	0.49	(0.67)	(0.77)	**2E-03**	**2E-03**	0.32	(0.57)	(0.73)
FRI	0.70	(0.74)	(0.83)	0.09	**8E-11**	0.60	(0.73)	(0.86)
FLC	0.59	(0.69)	(0.76)	0.98	**7E-09**	0.32	(0.52)	(0.68)

[a]Model: Phenotype $\sim X$.
[b]Model: Phenotype $\sim$ Latitude + FRI + X.
[c]Nominal p-value.
[d]Percentile of p-value compared to genome-wide SNP-based tests.
[e]Percentile of p-value compared to genome-wide haplotype-based tests.
