# Supplementary material for: An Arabidopsis Example of Association Mapping in Structured Samples
Source: PLoS Genet. 2007 Jan 19;3(1):e4. doi: 10.1371/journal.pgen.0030004 (PMC1779303; doi:10.1371/journal.pgen.0030004)
Supplement: Supplementary file 15 [file pgen.0030004.st003.pdf]

Table S3. Analysis of candidate polymorphisms using model: $\sim$ Latitude + FRI + X + X $\times$ Latitude.

			X			X $\times$ **Latitude**		
**Phenotype**	**Latitude**	**FRI**	**raw**[a]	**%SNP**[b]	**%HAP**[c]	**raw**[a]	**%SNP**[b]	**%HAP**[c]
				X = *CRY2* [32]				
LD	**6E-08**	**2E-07**	**2E-04**	(0.07)	(0.21)	**2E-03**	(**0.03**)	(0.07)
LDV	**6E-07**	**0.01**	**6E-04**	(0.07)	(0.20)	0.01	(0.07)	(0.15)
SD	**2E-04**	**6E-05**	**2E-03**	(0.07)	(0.22)	**0.04**	(0.16)	(0.29)
SDV	**3E-06**	0.12	**5E-04**	(**0.04**)	(0.11)	0.14	(0.30)	(0.40)
JIC0W	0.05	**5E-06**	**2E-03**	(**0.02**)	(0.06)	**0.02**	(0.05)	(0.07)
JIC2W	**2E-04**	**7E-06**	**3E-04**	(**0.05**)	(0.12)	0.02	(0.10)	(0.16)
JIC4W	**5E-05**	**5E-04**	**5E-04**	(0.06)	(0.18)	0.04	(0.19)	(0.31)
JIC8W	0.16	**0.01**	**1E-03**	(**0.03**)	(0.07)	0.09	(0.19)	(0.27)
±V (LD)	**1E-05**	**2E-07**	**4E-03**	(0.12)	(0.27)	**0.01**	(0.07)	(0.12)
±V (SD)	**0.03**	**3E-03**	0.37	(0.45)	(0.52)	0.80	(0.81)	(0.84)
SD/LD (V)	0.07	0.44	0.12	(0.24)	(0.37)	0.40	(0.52)	(0.66)
VERN	0.08	**2E-05**	0.42	(0.56)	(0.66)	0.05	(0.11)	(0.17)
JIC/USC	**0.02**	0.61	0.15	(0.23)	(0.30)	0.39	(0.38)	(0.41)
JIC/USC (V)	**7E-03**	**2E-03**	**0.01**	(0.11)	(0.28)	0.24	(0.48)	(0.65)
FRI	0.21	**1E-11**	**2E-03**	(**0.02**)	(**0.03**)	0.34	(0.45)	(0.52)
FLC	0.90	**8E-09**	0.73	(0.80)	(0.86)	0.23	(0.29)	(0.32)
				X = *FLC* [33]				
LD	**1E-08**	**2E-06**	0.52	(0.77)	(0.89)	**7E-02**	(0.25)	(0.40)
LDV	**1E-07**	**0.01**	0.98	(0.99)	(1.00)	0.18	(0.38)	(0.52)
SD	**1E-05**	**1E-04**	0.06	(0.30)	(0.51)	0.09	(0.24)	(0.38)
SDV	**5E-08**	0.16	**0.02**	(0.18)	(0.34)	0.24	(0.40)	(0.53)
JIC0W	**0.01**	**4E-05**	0.21	(0.37)	(0.52)	0.42	(0.54)	(0.63)
JIC2W	**2E-05**	**2E-05**	0.17	(0.42)	(0.59)	0.07	(0.22)	(0.34)
JIC4W	**3E-06**	**6E-04**	0.11	(0.37)	(0.58)	**0.04**	(0.19)	(0.31)
JIC8W	0.05	**0.02**	0.17	(0.35)	(0.51)	0.57	(0.66)	(0.77)
±V (LD)	**2E-06**	**1E-06**	0.49	(0.71)	(0.83)	0.11	(0.28)	(0.42)
±V (SD)	**0.01**	**3E-03**	0.36	(0.44)	(0.51)	0.66	(0.70)	(0.73)
SD/LD (V)	**0.01**	0.28	**1E-03**	(**0.01**)	(**0.02**)	0.97	(0.97)	(0.98)
VERN	0.05	**6E-05**	0.29	(0.43)	(0.55)	0.46	(0.52)	(0.62)
JIC/USC	**0.03**	0.58	**0.02**	(0.06)	(0.08)	0.93	(0.92)	(0.93)
JIC/USC (V)	**9E-04**	**1E-03**	**0.04**	(0.16)	(0.35)	**0.04**	(0.21)	(0.35)
FRI	0.05	**5E-10**	0.35	(0.48)	(0.60)	0.60	(0.67)	(0.74)
FLC	0.69	**3E-08**	0.16	(0.27)	(0.35)	0.65	(0.69)	(0.70)
				X = *PHYC* [34]				
LD	**1E-07**	**9E-07**	**0.01**	(0.22)	(0.44)	0.07	(0.25)	(0.39)
LDV	**9E-08**	**0.01**	0.50	(0.72)	(0.86)	0.90	(0.95)	(0.97)
SD	**1E-04**	**1E-04**	0.11	(0.38)	(0.59)	0.16	(0.33)	(0.48)
SDV	**1E-06**	0.14	0.07	(0.29)	(0.49)	0.35	(0.50)	(0.62)
JIC0W	**0.02**	**2E-05**	0.15	(0.30)	(0.46)	0.06	(0.12)	(0.17)
JIC2W	**8E-05**	**3E-05**	0.07	(0.27)	(0.45)	0.08	(0.25)	(0.38)
JIC4W	**7E-06**	**1E-03**	0.36	(0.63)	(0.81)	0.29	(0.51)	(0.69)
JIC8W	**0.04**	**0.02**	0.94	(0.96)	(0.98)	0.35	(0.47)	(0.57)
±V (LD)	**3E-05**	**3E-07**	**2E-03**	(0.09)	(0.22)	**0.01**	(0.07)	(0.13)
±V (SD)	**0.01**	**2E-03**	0.66	(0.71)	(0.80)	**5E-04**	(**2E-03**)	(**2E-03**)
SD/LD (V)	0.08	0.44	0.09	(0.20)	(0.31)	0.13	(0.25)	(0.35)
VERN	0.15	**1E-05**	**0.02**	(0.07)	(0.13)	**0.01**	(**0.04**)	(0.08)
JIC/USC	**0.03**	0.62	0.68	(0.72)	(0.79)	0.24	(0.23)	(0.26)
JIC/USC (V)	**2E-03**	**2E-03**	0.31	(0.53)	(0.70)	0.14	(0.36)	(0.54)
FRI	0.09	**9E-11**	0.60	(0.69)	(0.77)	0.44	(0.54)	(0.60)
FLC	0.98	**7E-09**	0.32	(0.43)	(0.53)	0.32	(0.39)	(0.42)

[a] Nominal p-value.
[b] Percentile of p-value compared to genome-wide SNP-based tests.
[c] Percentile of p-value compared to genome-wide haplotype-based tests.
