# Supplementary material for: An Arabidopsis Example of Association Mapping in Structured Samples
Source: PLoS Genet. 2007 Jan 19;3(1):e4. doi: 10.1371/journal.pgen.0030004 (PMC1779303; doi:10.1371/journal.pgen.0030004)
Supplement: Supplementary file 16 [file pgen.0030004.st004.pdf]

Table S4. Analysis of candidate polymorphisms using model: $\sim$ Latitude + FRI + X + $X \times$ Latitude + $X \times$ FRI.

			X			$X \times$ **Latitude**			$X \times$ **FRI**		
**Phenotype**	**Latitude**	**FRI**	**raw**[a]	**%SNP**[b]	**%HAP**[c]	**raw**[a]	**%SNP**[b]	**%HAP**[c]	**raw**[a]	**%SNP**[b]	**%HAP**[c]
					X = *CRY2* [32]						
LD	**6E-08**	**7E-09**	**7E-03**	(0.22)	(0.47)	**5E-03**	(0.06)	(0.10)	0.11	(0.10)	(0.11)
LDV	**5E-07**	**5E-04**	**4E-03**	(0.14)	(0.32)	**0.03**	(0.12)	(0.21)	0.08	(0.08)	(0.09)
SD	**2E-04**	**6E-06**	**0.03**	(0.24)	(0.49)	0.08	(0.22)	(0.35)	0.22	(0.18)	(0.17)
SDV	**2E-06**	**0.02**	**2E-03**	(0.06)	(0.17)	0.18	(0.33)	(0.43)	0.09	(0.08)	(0.09)
JIC0W	0.05	**2E-07**	0.09	(0.28)	(0.45)	**0.01**	(**0.05**)	(0.06)	0.80	(0.77)	(0.71)
JIC2W	**2E-04**	**2E-07**	**0.02**	(0.20)	(0.40)	**0.01**	(0.07)	(0.11)	0.73	(0.70)	(0.68)
JIC4W	**5E-05**	**1E-05**	**0.02**	(0.20)	(0.41)	**0.02**	(0.13)	(0.21)	0.83	(0.81)	(0.79)
JIC8W	0.16	**8E-04**	**0.01**	(0.09)	(0.19)	**0.05**	(0.12)	(0.19)	0.91	(0.90)	(0.90)
±V (LD)	**2E-05**	**3E-08**	0.07	(0.38)	(0.62)	**0.02**	(0.10)	(0.18)	0.31	(0.28)	(0.28)
±V (SD)	**0.03**	**7E-03**	0.18	(0.30)	(0.39)	0.71	(0.73)	(0.76)	0.64	(0.64)	(0.66)
SD/LD (V)	0.07	0.74	0.09	(0.21)	(0.32)	0.32	(0.45)	(0.55)	0.53	(0.56)	(0.60)
VERN	0.08	**2E-05**	0.66	(0.78)	(0.88)	0.05	(0.12)	(0.19)	0.57	(0.51)	(0.45)
JIC/USC	**0.01**	0.46	0.16	(0.23)	(0.30)	0.71	(0.69)	(0.70)	0.05	(**0.01**)	(**0.02**)
JIC/USC (V)	**7E-03**	**2E-04**	0.12	(0.34)	(0.55)	0.16	(0.38)	(0.55)	0.58	(0.50)	(0.46)
FRI	0.20	**1E-12**	**0.05**	(0.22)	(0.39)	0.17	(0.27)	(0.32)	0.43	(0.56)	(0.65)
FLC	0.90	**1E-08**	0.80	(0.88)	(0.93)	0.33	(0.37)	(0.40)	0.51	(0.59)	(0.64)
					X = *FLC* [33]						
LD	**1E-08**	**5E-06**	0.08	(0.46)	(0.68)	0.07	(0.24)	(0.37)	0.45	(0.45)	(0.45)
LDV	**1E-07**	**0.01**	0.54	(0.76)	(0.89)	0.18	(0.36)	(0.50)	0.46	(0.46)	(0.48)
SD	**1E-05**	**7E-04**	**0.01**	(0.16)	(0.38)	0.09	(0.24)	(0.39)	0.82	(0.81)	(0.78)
SDV	**6E-08**	0.38	**0.01**	(0.13)	(0.28)	0.25	(0.40)	(0.51)	0.54	(0.53)	(0.53)
JIC0W	**0.01**	**1E-04**	**0.04**	(0.19)	(0.32)	0.42	(0.52)	(0.61)	0.75	(0.70)	(0.63)
JIC2W	**2E-05**	**2E-04**	**0.02**	(0.17)	(0.37)	0.07	(0.21)	(0.32)	0.95	(0.95)	(0.95)
JIC4W	**4E-06**	**3E-03**	**0.02**	(0.20)	(0.41)	0.04	(0.19)	(0.28)	0.57	(0.53)	(0.51)
JIC8W	**0.05**	**0.04**	0.06	(0.20)	(0.35)	0.58	(0.66)	(0.75)	0.81	(0.77)	(0.74)
±V (LD)	**2E-06**	**4E-06**	0.08	(0.38)	(0.62)	0.11	(0.27)	(0.40)	0.51	(0.47)	(0.47)
±V (SD)	**0.02**	**2E-03**	0.80	(0.86)	(0.90)	0.65	(0.69)	(0.71)	0.56	(0.57)	(0.59)
SD/LD (V)	**0.01**	0.08	**3E-03**	(**0.02**)	(**0.04**)	0.94	(0.95)	(0.97)	0.11	(0.14)	(0.17)
VERN	0.05	**2E-04**	0.05	(0.20)	(0.36)	0.47	(0.53)	(0.63)	0.83	(0.80)	(0.76)
JIC/USC	**0.03**	0.92	**0.02**	(0.05)	(0.07)	0.92	(0.91)	(0.92)	0.19	(0.09)	(0.08)
JIC/USC (V)	**9E-04**	**8E-03**	**5E-03**	(0.10)	(0.27)	**0.04**	(0.20)	(0.33)	0.24	(0.15)	(0.13)
FRI	**0.05**	**2E-09**	**0.02**	(0.14)	(0.28)	0.57	(0.63)	(0.70)	0.10	(0.21)	(0.26)
FLC	0.69	**2E-07**	**0.01**	(0.08)	(0.14)	0.62	(0.65)	(0.67)	0.13	(0.21)	(0.27)
					X = *PHYC* [34]						
LD	**0.02**	**8E-07**	**0.02**	(0.29)	(0.55)	0.09	(0.27)	(0.41)	0.48	(0.49)	(0.48)
LDV	0.54	**0.01**	0.54	(0.76)	(0.89)	0.80	(0.88)	(0.92)	0.49	(0.48)	(0.52)
SD	0.14	**1E-04**	0.14	(0.46)	(0.68)	0.15	(0.31)	(0.46)	0.77	(0.76)	(0.74)
SDV	0.08	0.13	0.08	(0.31)	(0.52)	0.51	(0.62)	(0.71)	0.48	(0.48)	(0.47)
JIC0W	0.23	**1E-05**	0.23	(0.45)	(0.62)	0.03	(0.08)	(0.10)	0.94	(0.93)	(0.90)
JIC2W	0.13	**2E-05**	0.13	(0.41)	(0.59)	0.12	(0.29)	(0.42)	0.47	(0.43)	(0.39)
JIC4W	0.47	**9E-04**	0.47	(0.74)	(0.88)	0.30	(0.51)	(0.69)	0.76	(0.73)	(0.71)
JIC8W	0.86	**0.02**	0.86	(0.92)	(0.96)	0.45	(0.55)	(0.66)	0.58	(0.50)	(0.47)
±V (LD)	**3E-03**	**2E-07**	**3E-03**	(0.14)	(0.34)	**0.01**	(0.07)	(0.13)	0.48	(0.44)	(0.43)
±V (SD)	0.62	**2E-03**	0.62	(0.72)	(0.81)	**2E-03**	(**8E-03**)	(**0.01**)	0.11	(0.11)	(0.10)
SD/LD (V)	0.08	0.46	0.08	(0.20)	(0.30)	0.49	(0.60)	(0.70)	0.06	(0.08)	(0.10)
VERN	**0.02**	**9E-06**	**0.02**	(0.11)	(0.22)	**3E-03**	(**0.02**)	(**0.04**)	0.98	(0.98)	(0.97)
JIC/USC	0.66	0.63	0.66	(0.69)	(0.76)	0.08	(0.10)	(0.13)	0.58	(0.48)	(0.40)
JIC/USC (V)	0.40	**2E-03**	0.40	(0.64)	(0.78)	0.10	(0.31)	(0.47)	0.95	(0.93)	(0.92)
FRI	0.76	**1E-10**	0.76	(0.83)	(0.92)	0.66	(0.70)	(0.78)	0.44	(0.57)	(0.66)
FLC	0.42	**7E-09**	0.42	(0.61)	(0.75)	0.21	(0.24)	(0.29)	0.81	(0.84)	(0.88)

[a]Nominal p-value.
[b]Percentile of p-value compared to genome-wide SNP-based tests.
[c]Percentile of p-value compared to genome-wide haplotype-based tests.
